# Neutrophil Extracellular Vesicles and Airway Smooth Muscle Proliferation in the Natural Model of Severe Asthma in Horses

**DOI:** 10.3390/cells11213347

**Published:** 2022-10-24

**Authors:** Sophie Mainguy-Seers, Francis Beaudry, Christopher Fernandez-Prada, James G. Martin, Jean-Pierre Lavoie

**Affiliations:** 1 Department of Clinical Sciences, Faculty of Veterinary Medicine, Université de Montréal, St-Hyacinthe, QC J2S 2M2, Canada; 2 Department of Veterinary Biomedical Sciences, Faculty of Veterinary Medicine, Université de Montréal, St-Hyacinthe, QC J2S 2M2, Canada; 3 Department of Pathology and Microbiology, Faculty of Veterinary Medicine, Université de Montréal, St-Hyacinthe, QC J2S 2M2, Canada; 4 Meakins Christie Laboratories, Research Institute of the McGill University Health Centre, Montréal, QC H4A 3J1, Canada

**Keywords:** asthma, neutrophils, airway smooth muscle, extracellular vesicles, proteomics, equine

## Abstract

Extracellular vesicles (EVs) contribute to intercellular communication through the transfer of their rich cargo to recipient cells. The EVs produced by LPS-stimulated neutrophils from healthy humans and horses increase airway smooth muscle (ASM) proliferation, but the roles of neutrophil EVs in asthma are largely unexplored. The aim of this study was to determine whether neutrophil-derived EVs isolated during the remission or exacerbation of asthma influence ASM proliferation differentially. Peripheral blood neutrophils were collected during remission and exacerbation in eight horses affected by severe asthma. The cells were cultured (±LPS), and their EVs were isolated by ultracentrifugation and characterized by laser scattering microscopy and proteomic analysis. The proliferation of ASM co-incubated with EVs was monitored in real time by electrical impedance. Two proteins were significantly upregulated during disease exacerbation in neutrophil EVs (MAST4 and Lrch4), while LPS stimulation greatly altered the proteomic profile. Those changes involved the upregulation of neutrophil degranulation products, including proteases known to induce myocyte proliferation. In agreement with the proteomic results, EVs from LPS-stimulated neutrophils increased ASM proliferation, without an effect of the disease status. The inhalation of environmental LPS could contribute to asthma pathogenesis by activating neutrophils and leading to ASM hyperplasia.

## 1. Introduction

Asthma is a chronic and often debilitating respiratory disease affecting approximately 300 million people over the globe and causing over 400,000 deaths annually [1]. Although often characterized as an eosinophilic and allergic condition, a substantial subset of patients with asthma, especially in its severe form, present with airway neutrophilia [2,3]. Neutrophilic inflammation has been associated with several negative outcomes, including fatal episodes [4] and fixed airflow obstruction [5]. The latter is believed to be a consequence of chronic bronchial remodeling, particularly of the increased mass of the airway smooth muscle (ASM), which contributes to both fixed and dynamic obstruction through bronchospasm. Severe equine asthma is an incurable respiratory disease occurring in genetically predisposed horses and in which periods of clinical remission and exacerbation alternate in response to the degree of environmental antigenic exposure. The disease is characterized by expiratory airflow obstruction, airway neutrophilic inflammation, and bronchial remodeling, including an increase in the ASM mass [6]. Similar to asthma in humans, pulmonary neutrophilia is associated with the degree of airway obstruction and a poor response to corticosteroid therapy in horses with severe asthma [7]. Although equine asthma does not reproduce all the pathophysiological events occurring in humans, it is the only spontaneously occurring animal disease that shares many features with the severe neutrophilic form of asthma in humans. The large size of horses is particularly amenable to the collection of bronchial biopsies and the quantities of blood that allow for the isolation of peripheral neutrophils; therefore, the equine model was used in this study with the aim of obtaining results that could benefit both equine and human health.

The mechanisms responsible for the association of neutrophilic inflammation with the severity of human and equine asthma remain unclear. Neutrophilic proteases such as elastase [8] and matrix metallopeptidase 9 (MMP-9) [9] could contribute to the increase in ASM mass through their mitogenic effects. However, neutrophils are only rarely observed within the ASM [10], suggesting that a direct topical effect of neutrophil products is unlikely. Nevertheless, neutrophils could contribute to ASM remodeling through the release of extracellular vesicles (EVs). These nanoscale particles are produced by virtually all cell types and play significant roles in coagulation, intercellular communication and metabolic activity [11]. EVs can influence local and distant recipient cells that internalize their contents, which include, but are not limited to, nucleic acids, proteins, and lipids [11]. Importantly, EVs from lipopolysaccharide (LPS)-stimulated neutrophils from healthy humans and horses have been shown to be internalized by and to increase ASM proliferation [12,13].

This study aimed to determine whether the asthmatic disease status (clinical remission or exacerbation) alters the proteomic content and the biological effects of neutrophil EVs in asthma, using the natural model of severe asthma in horses. We hypothesized that EVs from neutrophils isolated during exacerbation would reflect the activated state of the parent cells, resulting in an enhanced proliferative effect on ASM (primary outcome). As endotoxins contribute to the development of airway neutrophilia and respiratory dysfunction in asthmatic humans [14,15] and horses [16,17] and are omnipresent in houses [18] and barns [19], we also investigated whether the treatment of neutrophils with LPS would enhance the biological effects and the protein content of neutrophil EVs in asthma.

## 2. Materials and Methods

### 2.1. Study Protocol

All experimental procedures were performed in accordance with the Canadian Council for Animal Care guidelines and were approved by the Animal Care Committee of the Faculty of Veterinary Medicine of the Université de Montréal on 10 February 2021 (Protocol # 21-Rech-2108). This manuscript follows the recommendations of the ARRIVE guidelines.

This experimental study was performed with eight horses with well-characterized severe asthma which were donated to a research herd. Remission or exacerbation of the disease was obtained by dietary and environmental management. Horses were first assessed when no clinical signs of asthma were present, while living in conditions known to maintain clinical remission (fed haylage and living outside (six horses) or fed oiled mixed hay [20] and living in the barn (two horses)). The exacerbation of the disease was induced by a natural antigenic exposure (by stabling and dry hay feeding, with daily access to turnout). The horses were evaluated after four to eight weeks of stabling, when respiratory distress was present at rest. At the end of the study, the horses were kept within the research herd, and clinical remission was re-acquired by dietary and environmental modifications.

### 2.2. Pulmonary Function Test

The Equine MasterScreen impulse oscillometry system (Jaeger GmbH, Würzburg, Germany) was used to measure lung function in unsedated horses with the head positioned physiologically, as previously reported [21]. Briefly, impulses produced by a loudspeaker were superimposed on the tidal breathing of the horse through an airtight mask. Simultaneously, a pneumotachograph connected to a pressure transducer and placed in front of the mask acquired the pressure-flow signal response of the respiratory system. The calibration of the device was performed on each test day, followed by a verification of the accuracy with a resistive test load. After data acquisition with LabManager (version 4.53, Jaeger, Würzburg, Germany), the analysis was completed with Fast-Fourier transform with the FAMOS software (IMC, Meβsysteme, Berlin, Germany). The results of three 30 s recordings were averaged for analyses. Impulse oscillometry measures impedance, which represents the forces opposing the sound wave imposed on the respiratory system and is composed of two parameters: pulmonary resistance and reactance. Resistance represents the resistive force opposing the airflow (mostly attributable to the central airways), while reactance is influenced by the elastic and inertance properties of the lung [22]. Higher and lower frequency impulses represent changes occurring in the upper/central and peripheral airways, respectively [23]. The whole breath resistance and reactance from 2 to 7 Hertz were analyzed.

### 2.3. Endoscopy and Collection of Respiratory Samples

Endoscopy was performed on sedated horses (detomidine (0.01 mg/kg, IV) or xylazine (0.3 mg/kg, IV), and butorphanol (0.02 mg/kg, IV)) with a 1.6 m video-endoscope (12.8 mm external diameter; Evis Exera II CV-180, Olympus Canada, Richmond Hill, ON, Canada). The bronchoalveolar lavages (BAL) were performed as previously described [24], with additional details in the online supplement. Endobronchial biopsies (7–10) were collected with smooth oval forceps (Standard Fenestrated and Smooth, 2.3 m, Olympus Medical Systems, Tokyo, Japan) after topical anesthesia (0.5% lidocaine hydrochloride) [25] during the remission sampling.

### 2.4. Neutrophil Isolation

Blood was drawn (160 mL) via jugular venipuncture into K_3_-EDTA tubes. After a 40 min sedimentation, the plasma-rich layer was recovered, and a density gradient centrifugation with Ficoll-PaqueTM Premium 1.084 (GE Healthcare Bio-sciences Corp., Mississauga, ON, Canada) was applied. The erythrocytes were lysed with distilled water, and the polymorphonuclear neutrophils were washed and suspended in a buffer solution (PBS 1X, EDTA 0.5 mM, BSA 0.2%). Cell concentration and viability were evaluated with the ADAM automatic Cell Counter (Montreal-Biotech Inc., Montreal, QC, Canada). Cytologic smears were stained with a modified Wright–Giemsa solution (Diff-Quik, Fisher Scientific, Hampton, NH, USA), and purity was determined by counting 400 cells. Neutrophils were resuspended at 5 × 10^6^ cells/mL in a culture medium (RPMI 1640 + L-glutamine supplemented with 10% low-endotoxin heat-inactivated fetal bovine serum (FBS, Wisent Inc., Saint-Jean-Baptiste, QC, Canada) depleted of EVs by overnight ultracentrifugation, 2 mM l-glutamine, 100 U/mL penicillin and 100 μg/mL streptomycin (all products from GIBCO, Life Technologies, Burlington, ON, Canada)) and cultured in six-well plates (non-treated plastic). Neutrophils were cultured with or without LPS (from *Escherichia coli* 0:111B4; 100 ng/mL, Sigma-Aldrich, St-Louis, MO, USA) for 18 h in a humidified incubator (37 °C, 5% CO_2_) to obtain vesicles produced spontaneously and from stimulated neutrophils. The supernatant from the neutrophil culture was centrifuged at 300× *g* for 10 min to remove cells and then at 2000× *g* for 10 min to remove dead cells; then, they were kept at −80 °C until the next steps of EVs isolation.

### 2.5. Neutrophil-Derived Extracellular Vesicles Isolation and Characterization

#### 2.5.1. Extracellular Vesicles Isolation

The culture supernatant was thawed and centrifuged at 10,000× *g* for 30 min to remove cell debris. Then, the supernatant was filtered through a 0.22 μm polyvinylidene fluoride membrane, and the EVs were pelleted by ultracentrifugation at 100,000× *g* for 60 min (Type 70Ti S/N O9U T301 fixed angle rotor in an Optima L-100 XP Beckman Coulter ultracentrifuge). The EVs pellet was resuspended in 15 mL of PBS and ultracentrifuged at 100,000× *g* for 60 min for a second time to remove potential protein contaminants. The pellet containing EVs was resuspended in PBS (≈500 µL; divided into aliquots and stored at −80 °C) for the functional assay and proteomic analysis.

#### 2.5.2. Nanoparticle Tracking Analysis

The EVs size and quantification were performed by laser scattering microscopy with the ZetaView PMX 120 device (Particle Metrix, Meerbusch, Germany) and software (version 8.05.11 SP4) as described by the manufacturer (details in the Supplement).

#### 2.5.3. Proteomics of Neutrophil Extracellular Vesicles

The protein content of the neutrophil EVs was analyzed in six horses that had adequate material in all four conditions. Protein digestion and mass spectrometry experiments were performed by the Proteomics platform of the CHU de Québec Research Center, Québec, Canada. For protein identification, mass spectra were searched against the Uniprot *Equus caballus* database (UniProt Reference Proteome—Proteome ID UP000002281—44,484 entries—2021.05) using the search engine Andromeda integrated into the MaxQuant software (https://www.maxquant.org/, version 2.0.2.0, accessed on 14 September 2022). The details of the proteomic methodology and statistical analysis are provided in the online supplement.

### 2.6. Airway Smooth Muscle Isolation and Characterization

#### 2.6.1. Cell Culture

The endobronchial biopsies collected from each horse were kept in transport media at 4 °C until processing within 60 min. Enzymatic digestion was performed as previously described [26]. The ASM cells were passaged at a 1:3 ratio with trypsin when they reached ≈80% confluence (every ~7 days, as expected for these cells [26]). The cells displayed the typical hill-and-valley appearance and spindle morphology of ASM cells [26], except for one horse, in which multiple areas of vacuolized cells and poor cell membrane definition were observed; this sample was discarded. For this subject, the co-culture with EVs was performed with the ASM from another asthmatic horse. At passage (P) 2, the cells were collected and slowly frozen using a commercial freezing container (ThermoFischer Scientific) for 48 h and then kept in liquid nitrogen until used. Detailed culture conditions are provided in the online supplement.

#### 2.6.2. Flow Cytometry

The purity of each ASM cell culture at the end of P4 was assessed by flow cytometry using specific contractile proteins (anti-smooth muscle α-actin (α-SMA) and anti-desmin), as previously described (details in the online supplement) [26].

#### 2.6.3. Airway Smooth Muscle Cells’ Real-Time Proliferation Assay

The cells at P5 were used for the proliferation assay in the Xcelligence device, as previously described, with minor modifications [12]. The background impedance in each well was measured using 40 μL of the complete medium. Then, 14,000 ASM cells were seeded in 100 μL of the complete medium in each well and left to equilibrate at room temperature for 30 min before data recording. The ASM cells were grown at 37 °C in a humidified atmosphere with 5% CO_2_ for 20 h and monitored for impedance to assess baseline cell proliferation. Autologous extracellular vesicles from each condition (vesicles from neutrophils collected in remission or exacerbation, treated or not treated with LPS) were resuspended in 60 μL of culture media (4000 EVs/ASM cell) and added after the first 20 h of the culture, when proliferation was no longer exponential (plateau-phase cells), as previously described [12]. Three types of control were used: negative (no cells, only culture medium), control with untreated ASM cells (cells with the culture medium), and ASM cells incubated with the supernatant from the last ultracentrifugation step of EVs isolation (to determine if residual LPS, if present, could influence ASM proliferation). In each condition, the same amount of culture medium and FBS was added to ensure comparable conditions. Each condition was performed in triplicate. The impedance value of each well was monitored every 15 min for a total duration of 50 h. The data were analyzed using the Xcelligence software (version 1.2.1) and expressed as the impedance normalized to the last cell index recorded before the time of EV addition (mean of the triplicates). The rate of cell growth was determined by calculating the slope of the line between the time point 20 h (just before the addition of EVs) and the time point 50 h. The data are shown starting at 15 h after the addition of EVs, when electrical impedance stabilized among the triplicates.

### 2.7. Statistical Analysis

The data were analyzed using GraphPad Prism version 9.3.1 for Windows (GraphPad Software, San Diego, CA, USA). Normality was assessed with the Shapiro–Wilk test. Data are shown with the mean and standard error of the mean when normally distributed and with the median and 25th and 75th percentile when not. BAL fluid percentages were compared with paired T-tests, or Wilcoxon tests, as appropriate. Two-way ANOVA with Bonferroni multiple comparison tests was used for the rest of the data (with the disease status and the cell treatment with or without LPS as independent variables for most analyses, and with the disease status and the impulse frequency as independent variables for lung function evaluation).

## 3. Results

### 3.1. Characterization of the Clinical Status of Horses with Severe Asthma

**Figure 1:**
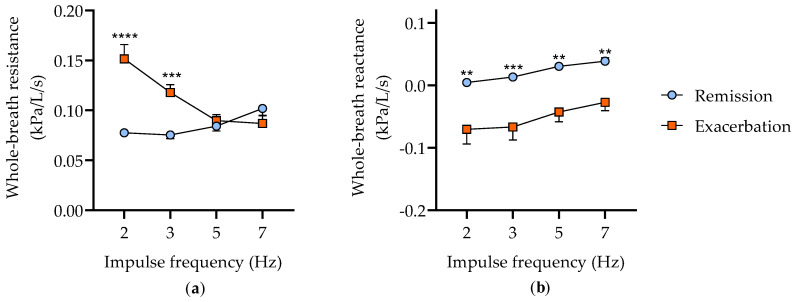
Lung function measured by oscillometry. Whole-breath resistance (**a**) and reactance (**b**) during remission and exacerbation of severe equine asthma (mean ± SEM). ** *p* < 0.01, *** *p* < 0.001, **** *p* < 0.0001 between remission and exacerbation with Bonferroni multiple comparison tests.

Eight horses with well-characterized severe asthma were studied (aged 16.9 ± 2.0 years, mixed breeds, five mares and three geldings). The horses were first evaluated during disease remission, when no clinical signs of asthma were present, and after several weeks of antigenic exposure, when respiratory efforts were visible at rest, indicating the development of an exacerbation. As expected, the whole-breath resistance was significantly increased (*p* < 0.0001) during clinical exacerbation, particularly at low impulse frequencies, demonstrating peripheral airway obstruction (effect of the impulse frequency *p* = 0.02, interaction between impulse frequency and disease status *p* < 0.0001; Figure 1a). Similarly, the whole-breath reactance was significantly decreased (*p* < 0.0001), mainly at lower impulse frequencies (*p* = 0.03) after the antigenic exposure (Figure 1b).

**Figure 2:**
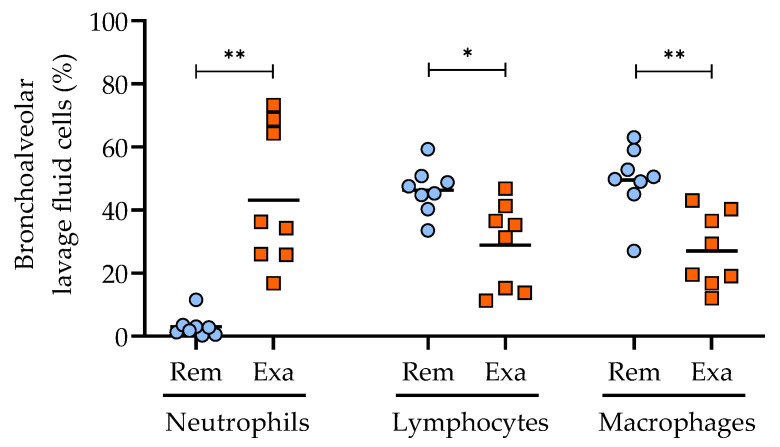
Percentages of inflammatory cells in the bronchoalveolar lavage fluid cytology of horses during remission (Rem) and exacerbation (Exa) of severe equine asthma (dot plot with mean). * *p* < 0.05, ** *p* < 0.01 with paired T-tests or Wilcoxon tests.

The clinical status was also confirmed by the cytology of BAL fluid, as horses with severe asthma are expected to develop substantial airway neutrophilia during exacerbation. The natural antigenic exposure with hay increased the airway neutrophilia in all horses (*p* = 0.008), with a concomitant decrease in the percentages of lymphocytes (*p* = 0.014) and macrophages (*p* = 0.003; Figure 2). The percentages of mast cells and eosinophils were low and did not vary with the clinical status (data not shown; the complete datasets are provided in the UdeM Dataverse repository).

### 3.2. Neutrophil-Derived Extracellular Vesicles

#### 3.2.1. Peripheral Blood Neutrophils Isolation

Once the clinical status had been characterized, blood neutrophils were isolated from each horse during disease remission and exacerbation by density gradient centrifugation. The mean neutrophil count per isolation was 332 (±33) million. The neutrophil purity was 98.7% (±0.2) in remission and 99.1% (±0.2) in exacerbation (*p*-NS). The median viability was 98% (97–99) and 98.5% (97–99) in remission and exacerbation, respectively (*p*-NS). These results were considered adequate for pursuing the isolation of neutrophil EVs.

#### 3.2.2. Nanoparticle Tracking Analysis

**Figure 3:**
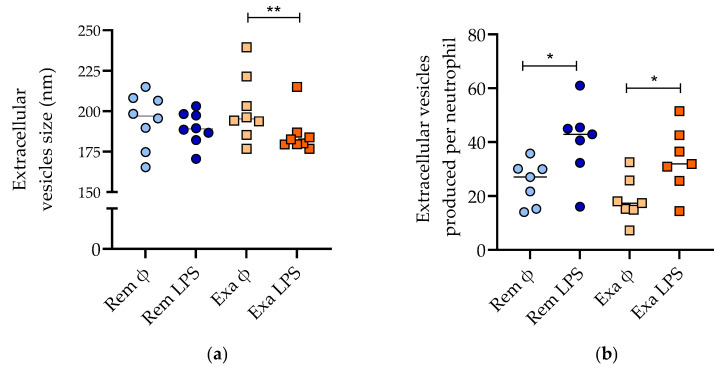
Neutrophil extracellular vesicle characterization by laser scattering microscopy. Size (**a**) and production per neutrophil (**b**) during remission (Rem) and exacerbation (Exa) (dot plot with mean). ϕ: untreated cells; LPS: cells treated with 100 ng/mL LPS. * *p* < 0.05, ** *p* < 0.01 with Bonferroni multiple comparison tests.

After the isolation of neutrophil-derived EVs by the ultracentrifugation and filtration of the culture supernatant, the EVs were characterized by laser scattering microscopy. The mean size of the EVs collected from the neutrophils (≈200 nm) was similar to that in previous reports [27]. There was an interaction between the disease status and the treatment of neutrophils with LPS (*p* = 0.04), as EVs produced by LPS-treated neutrophils were smaller, but only significantly during disease exacerbation (Figure 3a). There were more Evs produced when neutrophils were stimulated with LPS (*p* = 0.0002), without an effect of the disease status (Figure 3b).

#### 3.2.3. Proteomic Analysis of Neutrophil Extracellular Vesicles

The protein content of the EVs was studied in six horses in which sufficient material was available in all four conditions (the neutrophils were isolated during remission or exacerbation, treated or not treated with LPS). Mass spectrometry analysis allowed for the identification of 1403 proteins across all conditions. The differential regulation of proteins was compared between the disease statuses and between the untreated and LPS-stimulated cells. Two proteins were regulated by the disease status; leucine-rich repeats and calponin homology domain-containing 4 (Lrch4) and microtubule-associated serine/threonine-protein kinase 4 (MAST4) were upregulated during asthma exacerbation.

**Figure 4:**
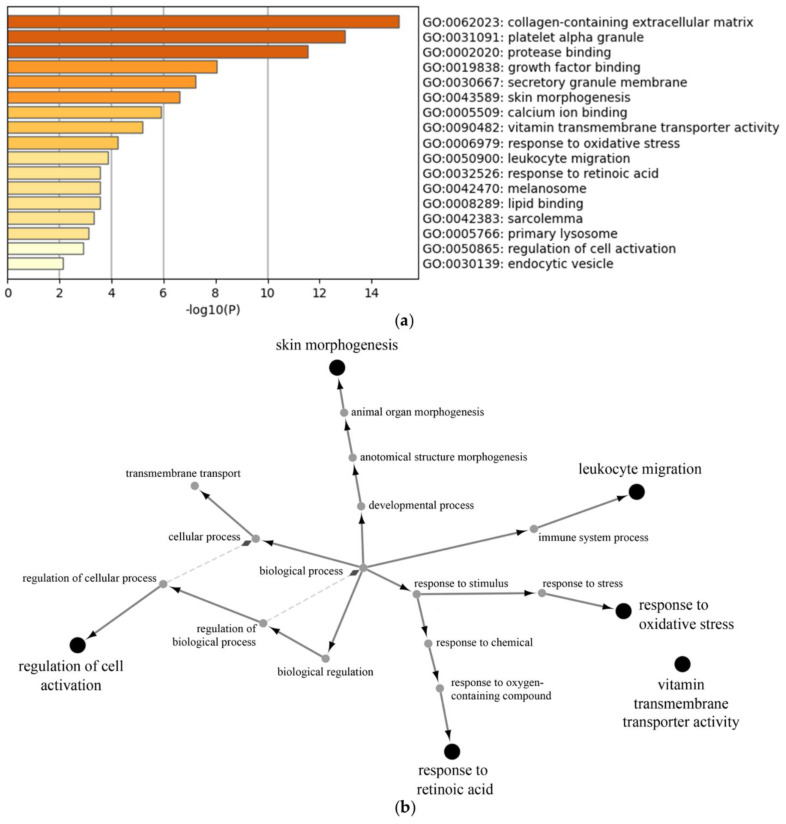
Gene ontology enrichment analysis of proteins downregulated by LPS in neutrophil-derived EVs. Enrichment clusters identified by Metascape (**a**) and functional analysis of enriched GO terms from the parent node to the root node, illustrated with ClueGo and Cluepedia in Cytoscape (**b**).

**Figure 5:**
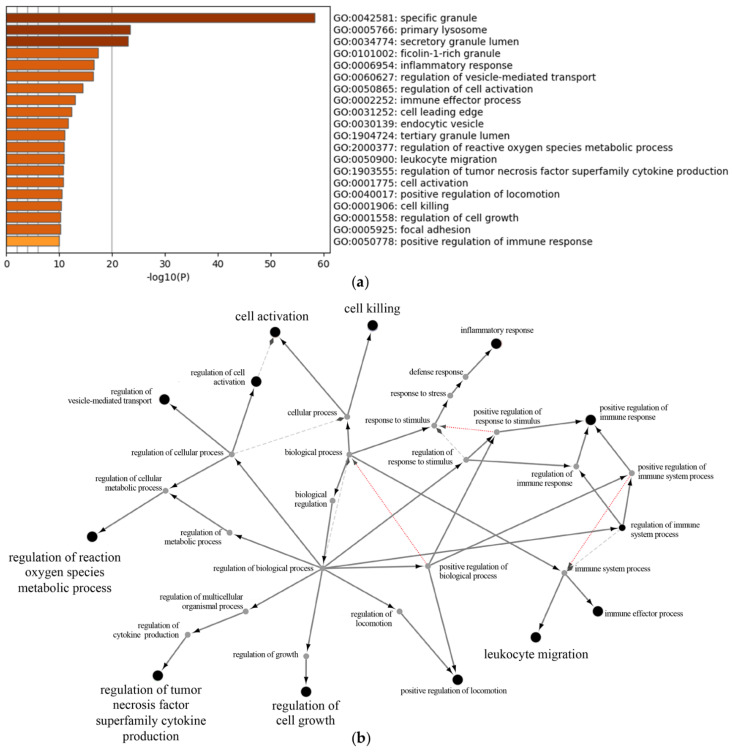
Gene ontology enrichment analysis of proteins upregulated by LPS in neutrophil-derived EVs. Enrichment clusters identified by Metascape (**a**) and functional analysis of enriched GO terms from the parent node to the root node, illustrated with ClueGo and Cluepedia in Cytoscape (**b**).

**Figure 6:**
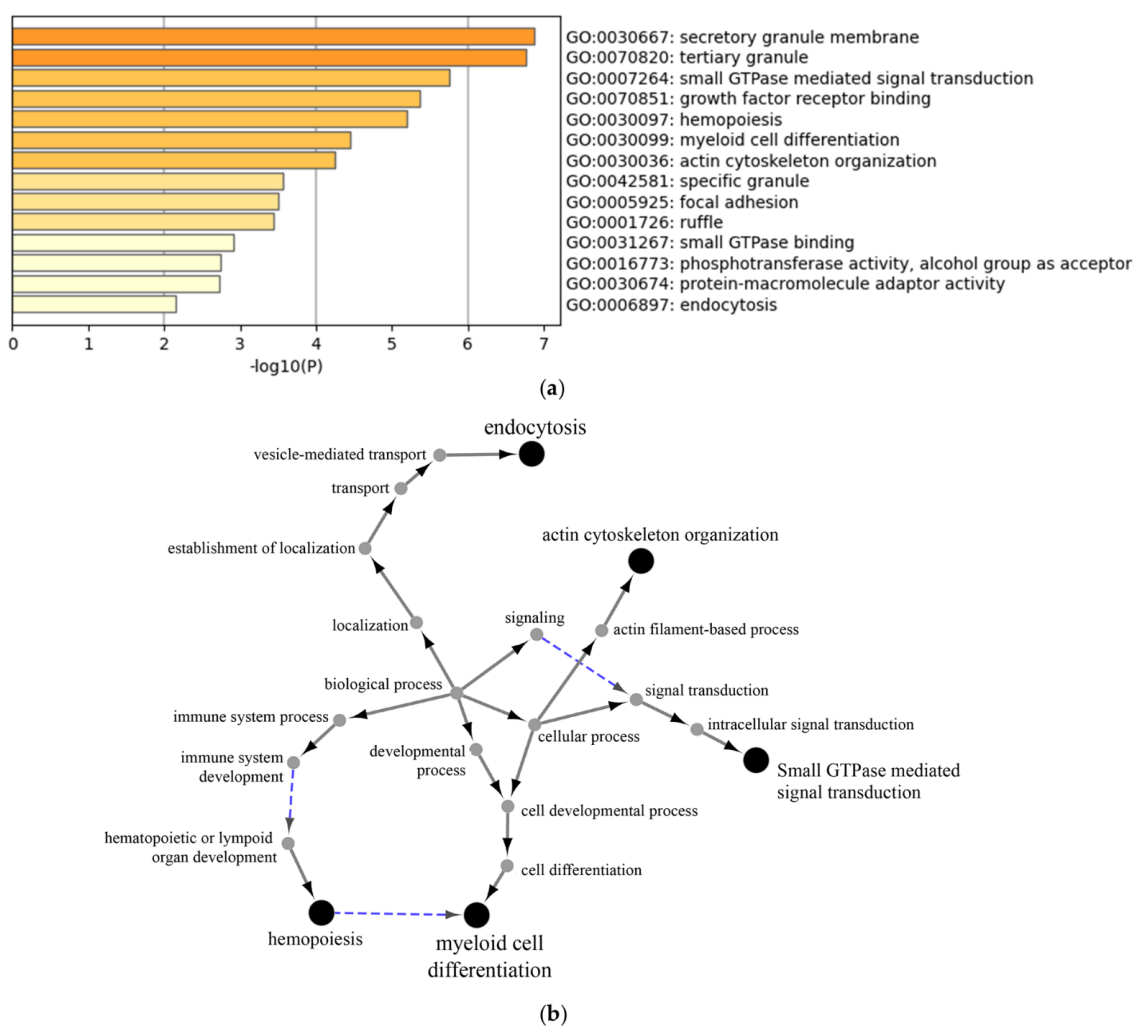
Gene ontology enrichment analysis of proteins upregulated by LPS in neutrophil-derived EVs only during asthmatic exacerbation. Enrichment clusters identified by Metascape (**a**) and functional analysis of enriched GO terms from the parent node to the root node, illustrated with ClueGo and Cluepedia in Cytoscape (**b**).

The stimulation of neutrophils with LPS resulted in the downregulation of 38 proteins and the upregulation of 172 proteins (Supplementary Tables S1 and S2). There were 25 proteins in which the LPS stimulation caused an upregulation solely during disease exacerbation (Supplementary Table S3). Gene ontology analysis using Metascape allowed for the determination of the biological processes altered by LPS (downregulation by LPS (Figure 4a), upregulation by LPS (Figure 5a), upregulation by LPS during disease exacerbation only (Figure 6a) and Supplementary Table S4, which shows the proteins involved in each biological process). These included the regulation of neutrophil degranulation products, the regulation of cell growth and activation, and the regulation of vesicle-mediated transport. The protein–protein interaction networks of the biological processes altered by LPS were visualized with the ClueGo and Cluepedia plugins in the Cytoscape software (downregulation by LPS (Figure 4b), upregulation by LPS (Figure 5b), upregulation by LPS during disease exacerbation only (Figure 6b)). The analyses performed with the Reactome database are provided in the supplement (Supplementary Figures S1–S3).

### 3.3. Proliferative Effects of Extracellular Vesicles on Airway Smooth Muscle Cells

#### 3.3.1. ASM Characterization

The percentages of positive cells for α-SMA and desmin were, respectively, 99.7% (98.4–99.9) and 78.4% ± 4.9, confirming the selective culture of ASM cells isolated from the asthmatic horses (Supplementary Figure S4) [26].

#### 3.3.2. ASM Proliferation

**Figure 7:**
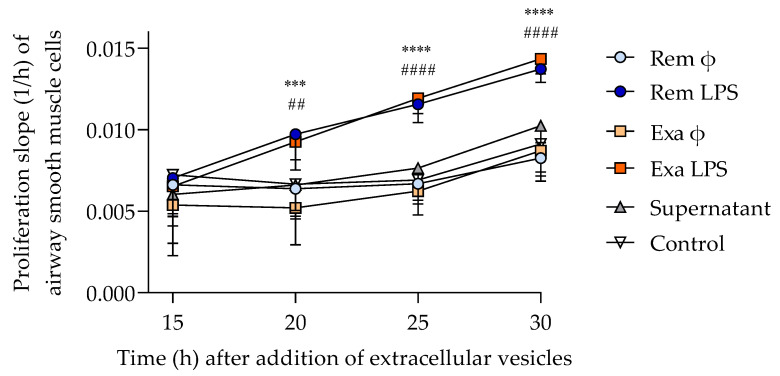
Airway smooth muscle proliferation studied with the Xcelligence device (mean ± SEM). ϕ: untreated cells; LPS: cells treated with 100 ng/mL of LPS. *** *p* < 0.001, **** *p* < 0.0001 between REM (remission) + LPS and control; ## *p* < 0.01, #### *p* < 0.0001 between EXA (exacerbation) + LPS and control with Bonferroni multiple comparison tests.

The proliferative effects of neutrophil EVs on ASM cells were studied in real time by electrical impedance. The number of remaining EVs was insufficient to perform the co-culture in one horse; therefore, seven subjects were used for this experiment. In each well, ASM cells were co-incubated with a fixed quantity of EVs (4000 EVs/cell) to ensure comparable conditions. The EVs produced by LPS-treated neutrophils significantly increased ASM proliferation (*p* = 0.0001), starting 20 h after co-incubation and lasting until the end of the experiment at 30 h, while the co-incubation with EVs from untreated neutrophils did not influence proliferation compared to the control wells (Figure 7). The proliferation of ASM cells incubated with the remaining supernatant from the last ultracentrifugation step of EVs isolation (to assess if residual LPS could alter the results) was not different from those of the control wells, indicating that remaining LPS, if there was any, was not responsible for the proliferative effects in EVs-treated ASM cells. The clinical status of the horses during neutrophil isolation (remission or exacerbation of asthma) did not influence the proliferation of ASM.

## 4. Discussion

Neutrophilic inflammation is associated with asthma severity and mortality; yet, the mechanisms underlying these negative outcomes are poorly understood. Extracellular vesicles are increasingly recognized as key mechanistic players in disease pathophysiology, including asthma [28]. The neutrophil activator LPS [29] greatly influenced the EV proteomic content in the current study, which was manifested by their pro-proliferative effect on ASM cells, as previously described [12]. However, the disease status (remission or exacerbation of severe equine asthma) did not influence the proliferative effect of neutrophil EVs, refuting the initial hypothesis.

This latter finding was unexpected, as the degree of neutrophil activation is enhanced in humans [30] and horses [31] affected by asthma. Indeed, the peripheral blood neutrophils from asthmatic patients produce more reactive oxygen species compared to healthy individuals, and more so when asthma is uncontrolled [32,33]. Additionally, the activation markers CD11b and CD35 are increased in the peripheral blood neutrophils from patients with corticosteroid-resistant severe asthma compared to those affected by a milder form and to healthy individuals [34]. Similarly, neutrophils from horses with asthma have increased respiratory burst activity after an antigenic challenge [35]. However, only two proteins in neutrophil EVs, Lrch4 and MAST4, were significantly upregulated during clinical exacerbation in the current study. Leucine-rich repeats and calponin homology domain-containing 4 (Lrch4) is a regulatory protein involved in toll-like receptors (TLR) signaling, including the classical LPS receptor TLR4, and it is involved in the production of cytokines (TNF-α and granulocyte-colony-stimulating factor) in response to LPS [36]. Importantly, the pulmonary knockdown of Lrch4 in mice reduces neutrophilic infiltration in response to LPS exposure, and it was recently proposed as a therapeutic target in inflammatory disease [36]. This finding merits attention in future studies, as TLR4 gene expression and LPS levels are increased in the sputum of neutrophilic asthmatics [37]. The other protein upregulated during asthma exacerbation was the estrogen-responsive protein microtubule-associated serine/threonine kinase family member 4 (MAST4). Although it has mainly been associated with central nervous system diseases and tumors, MAST4 is also related to asthma pathogenesis. In patients with mild asthma, its gene expression increases in the sputum after an aerosol allergen bronchoprovocation [38], and computational analysis revealed that MAST4 methylation is involved in the regulation of ASM response to interleukins [39]. Curiously, its gene expression is also upregulated in bronchoalveolar lavage fluid cells from horses with mastocytic, but not with neutrophilic, airway inflammation [40]. As the horses in this latter study were free of respiratory signs, the results are not directly comparable to the current report.

In addition to studying spontaneously produced EVs, the stimulation of neutrophils with LPS was performed, as its level is correlated with airway neutrophilia in asthmatic children [41] and adults [37] and is inversely correlated with lung function [37]. Similarly, the airway obstruction is exacerbated by the inhalation of LPS in severe equine asthma [17]. The increased production of EVs and the alteration of the proteomic signature by LPS, including an upregulation of degranulation products and of pro-inflammatory cytokine release, underline the expected activation of neutrophils by this stimulus. The results of this study are in agreement with, and extend, the previous report that EVs from LPS-treated neutrophils have a pro-proliferative effect by providing several potential mechanistic explanations. Multiple neutrophil biological processes upregulated by LPS involved the granular content (including elastase, cathepsin G, MMP-9, and lipocalin-2). The proteinase elastase is of particular interest, as it is correlated with the degree of airway obstruction and neutrophilia in asthmatic humans [42] and horses [35]. Importantly, elastase is mitogenic for ASM cells through the activation of the extracellular signal-regulated kinase (ERK) pathway [8]. The tertiary granule protein MMP-9 also increases the proliferation of ASM [9] and is found in higher concentrations in the sputum of uncontrolled asthmatics [43]. Similarly, its protein concentration is higher in the lungs of horses with SEA [44] and is further elevated after the inhalation of LPS, more so in asthmatic horses [45]. The gelatinase-bound protein lipocalin-2 (NGAL) is associated with the degree of respiratory obstruction in asthma [46] and can induce ASM cell proliferation [47]. Lipocalin-2 is also elevated in the respiratory secretions of horses with severe asthma [44] and is further increased in lung tissue after an antigenic exposure [48]. Other biological processes stimulated by LPS that could have contributed to ASM proliferation include the regulation of the reactive oxygen species metabolic process (through the protein 5-lipoxygenase [49]) and the regulation of the tumor-necrosis-factor superfamily [50]. The proteins that are regulated by LPS and were previously shown to be implicated in asthma and in ASM proliferation also include the upregulation of matrix-metalloproteinase-1 (MMP-1) [51] and Abelson interactor 1 (Abi1) [52] and the downregulation of insulin-like growth factor-binding protein 2 (IGFBP-2) [53]. In the current investigation, the proteomic content of neutrophil EVs was explored to identify the potential biological processes involved in ASM proliferation; future mechanistic studies should aim to confirm which neutrophil proteins are responsible for this effect by using specific inhibitors, but the remaining number of EVs was insufficient to pursue such functional assays in the current study.

As peripheral blood neutrophils in asthma are increasingly activated, we postulated that LPS stimulation would enhance the difference in the proteomic content and the biological effects of EVs between the remission and exacerbation status. However, the clinical exacerbation had a marginal effect on neutrophil-derived EVs, limited to an interaction with LPS regarding their size and the expression of some proteins, without an impact on ASM proliferation. Indeed, LPS stimulation decreased the size of EVs, but only significantly so when horses were undergoing exacerbation of the disease. This could be explained by the diminution of the proportion of apoptotic bodies, the largest form of EVs, as both LPS [54,55] and an antigenic challenge in asthmatic horses [35] reduce neutrophil apoptosis. The fact that some proteins were only upregulated by LPS during disease exacerbation suggests that peripheral neutrophils might have been primed by the antigenic exposure, and several of these proteins were previously associated with asthma pathogenesis (including ADAM metallopeptidase domain 17 [56], formin-like protein 1 [57] and myristoylated alanine-rich C-kinase substrate (MARCKS)). The latter is a key protein in the regulation of mucus secretion by epithelial cells and in the degranulation and the migration of neutrophils [58]. Interestingly, the biological process of endocytosis emerged in the proteins upregulated by LPS only during exacerbation, with the protein–protein network pointing to the involvement of vesicle-mediated transport. Although this was not reflected by an increased proliferation of ASM cells, it could have other biological effects not examined in the current study. Indeed, the proteins included in the biological process of endocytosis are implicated in neutrophil chemotaxis (DOCK2 [59], LYN [60]) and myelopoiesis (GRB2) [61], which might contribute to the perpetuation of pulmonary inflammation by neutrophil EVs.

One limitation of this study is the lack of control horses, precluding the evaluation of the effect of the disease itself on the EV content and activity, although the objective of the current study was to evaluate the impact of the allergenic challenge on neutrophil vesicles in asthmatic subjects. The regulation of proteins by LPS in asthmatic horses differed somewhat from previous results obtained in healthy animals [12]. While lactotransferrin was upregulated in both studies, some proteins were upregulated by LPS in diseased horses but downregulated in healthy ones (lipocalin-2, complement C3, and integrin-β2), and vice versa (annexin A7 and fibrinogen A-α chain). The disease status and methodological differences in the proteomic technology could have contributed to those results. Another limitation relates to the lack of standardization in the literature concerning the optimal quantity of EVs to perform functional assays, and the number of neutrophil EVs likely to reach ASM cells through the systemic circulation or from local neutrophils is unknown. However, as neutrophils are the most abundant leukocytes in circulation and are markedly increased in the lung of neutrophilic asthmatics, their EVs could have substantial biological effects. Whether circulating neutrophil EVs are increased in asthmatics, as other types of microvesicles are [62], remains to be determined. This study focused on the mitogenic effects of EVs due to the importance of ASM hyperplasia in asthma [63], but their impacts on other ASM biological functions, such as migration and contraction, require further investigations. Whether other EV components contributed to the ASM proliferation (such as microRNA and mRNA) would also merit attention. Finally, studying airway neutrophils would have been of interest, as they might behave differently than circulating cells. Indeed, the biological functions of neutrophils might change during endothelial transmigration [64], which could consequently alter the content of their extracellular vesicles. However, predicting the changes occurring in EVs from airway neutrophils in asthma would be hazardous, as there are contradictory reports of increased [65] or decreased [66] activation of sputum neutrophils compared to polymorphonuclear cells in human asthmatics. Furthermore, it is not currently possible to isolate airway neutrophils with sufficient purity in horses [67], and while studying the biological functions of EVs in BAL fluid is a promising area of research, it would not permit the uncovering of the specific roles of EVs derived from neutrophils.

In conclusion, LPS-stimulated neutrophils release EVs with a pro-proliferative effect on ASM cells in horses with severe asthma, but an antigenic exposure did not enhance their biological activity. The absence of an accentuated proliferative effect of the vesicles during the exacerbation of the disease in the equine species does not exclude that such a phenomenon could occur in humans, in which the triggers of an asthma crisis are more numerous and heterogeneous. As many more neutrophils reside in the airways of human and equine asthmatics in the neutrophilic phenotype, these cells could still contribute to ASM hyperplasia by the production of bioactive EVs when exposed to LPS, a contaminant widely retrieved in the environment. The diagnostic potential of Lrch4 and MAST4 as biomarkers and the involvement of these proteins in asthma pathophysiology require further investigation.

## Data Availability

The datasets generated in the current study are available in the UdeM Dataverse repository (https://doi.org/10.5683/SP3/UEWRWP, accessed on 14 September 2022).

## Supplementary Materials

The following supporting information can be downloaded at: https://www.mdpi.com/article/10.3390/cells11213347/s1, Supplementary methods, Figure S1: Reactome enrichment analysis of proteins downregulated by LPS in neutrophil-derived EVs; Figure S2: Reactome enrichment analysis of proteins upregulated by LPS in neutrophil-derived EVs; Figure S3: Reactome enrichment analysis of proteins upregulated by LPS in neutrophil-derived EVs only during asthmatic exacerbation; Figure S4: ASM cells flow cytometry; Table S1: Proteins significantly downregulated in EVs produced by LPS-stimulated neutrophils; Table S2: Proteins significantly upregulated in EVs produced by LPS-stimulated neutrophils; Table S3: Proteins significantly upregulated in EVs produced by LPS-stimulated neutrophils, only during asthmatic exacerbation; Table S4: Biological processes enriched in proteins from neutrophil-derived EVs stimulated with LPS [68,69,70,71,72,73,74].
