# Supplementary material for: Neutrophil Extracellular Vesicles and Airway Smooth Muscle Proliferation in the Natural Model of Severe Asthma in Horses
Source: Cells. 2022 Oct 24;11(21):3347. doi: 10.3390/cells11213347 (PMC9653818; doi:10.3390/cells11213347)
Supplement: Supplementary file 1 [file cells-11-03347-s001.zip › cells-1946062-supplementary.pdf]

Supplement to: Neutrophil extracellular vesicles and airway smooth muscle proliferation in the natural model of severe asthma in horses

Sophie Mainguy-Seers, Francis Beaudry, Christopher Fernandez-Prada, James G. Martin and Jean-Pierre Lavoie

Supplementary methods

Experimental procedures

Animals

The neutrophil EVs from eight horses with severe asthma were compared between remission and exacerbation of the disease. No data were available for power analysis, but eight horses were considered sufficient as previous studies demonstrated differences in peripheral neutrophil biology after an antigenic challenge by using six horses with severe asthma [1,2]. All procedures on animals were performed at a research farm, where they are housed all year round. Horses were conditioned to stand in a stock and to wear a facemask for lung function measurements. The horses were monitored daily, and humane endpoints included reduced appetite, abnormal fecal output, hyperthermia, colic, respiratory distress, or any other medical conditions that could have interfered with the study. No adverse events occurred during the experiment and clinical remission was re-obtained at the end of the study by dietary and environmental modifications.

Endoscopic bronchoalveolar lavage

With endoscopic guidance, two boluses of 250 mL of warm sterile isotonic saline were sequentially instilled into a main bronchus and aspirated via a suction pump. The samples were kept on ice until processing within 60 minutes. A modified Wright–Giemsa solution (Diff-Quik, Fisher Scientific, Waltham, Massachusetts, USA) was used to stain cytocentrifuged preparations of BALF. Differential cell counts were performed blindly from 400 leukocytes. In horses, BALF differential cell counts are preferred to the total counts as the BALF volume recovered depends on the degree of airway obstruction [3].

Extracellular vesicles characterization

Nanoparticle tracking analysis

The ZetaView device allows the evaluation of the particle size and quantification by tracking and videorecording the movement of individual EVs by using Brownian motion. The instrument was used as previously described with minor modifications [4]. Briefly, the device evaluated 11 different positions throughout the cell for each sample, with two readings at each position. The device software provided the EVs concentration and size (mean, median, and 10th and 90th percentile diameter) of the sample after automated removal of outlier positions. The pre-acquisition parameters were set to a sensitivity of 79, a shutter speed of 96 and a frame rate of 30 frames per second. Post-acquisition parameters were set to a maximum size of 1000 pixels, a minimum size of 10 pixels and a minimum brightness of 25.

Sample preparation for proteomic analysis: protein extraction and digestion

Protein digestion and mass spectrometry experiments were performed by the Proteomics platform of the CHU de Québec Research Center, Québec, Canada. Solubilization of membranes was performed with the addition of sodium deoxycholate (DOC; final concentration of 1%) and ultrasonication on a Bioruptor (high intensity, 15 cycles of 30s on/off, Diagenode). Precipitation of proteins was carried out by adding five volumes of cold acetone (-20°C) for overnight incubation. After a 15-minute centrifugation at 10,000× *g*, proteins from the dry pellet were resuspended in 50 mM ammonium bicarbonate and 1% DOC and quantified with a Bradford assay (580 nm, Biorad). Protein denaturation prior to digestion was done by heating at 95°C for 5 min, cysteine disulfide bond reduction with dithiotreitol (0.2 mM at 37°C for 30 min) and cysteine alkylation with iodoacetamide (0.8 mM at 37°C for 30 min). Protein digestion was obtained by overnight incubation at 37°C with trypsin (1:50 protease:protein ratio, sequencing grade, Promega, Madison, WI). A solution of acetonitrile 3 % (ACN) trifluoric acid 1 % (TFA) and acetic acid 0.5% was used to stop the digestion. Tryptic peptides were desalted on C18 StageTips (Empore), vacuum dried and stored at -20°C prior to mass spectrometry analysis.

Mass spectrometry

Samples were resuspended in 2% ACN;0.05% TFA in water and their concentration adjusted with 205 nm absorbance readings (Nanodrop, Thermo Fischer) for the injection of 1 µg of samples on the liquid chromatography system. Samples were analyzed by nanoLC/MSMS using a Dionex UltiMate 3000 nanoRSLC chromatography system (Thermo Fisher Scientific) coupled to an Orbitrap Fusion mass spectrometer (Thermo Fisher Scientific, San Jose, CA,USA) equipped with a nanoelectrospray ion source. Peptides were trapped at 20µL/min in loading solvent (2% ACN, 0.05% TFA) on a 5 mm x 300 µm C18

pepmap cartridge (Thermo Fisher Scientific) for 5 minutes. Then, the pre-column was switched online with a separation column of 50 cm x 75 μm internal diameter (Pepmap Acclaim column, ThermoFisher) and the peptides were eluted with a linear gradient from 5-40% solvent B (A: 0.1% formic acid, B: 80% acetonitrile, 0.1% formic acid) in 90 minutes, at 300 nL/min. Mass spectra were acquired using a data-dependent acquisition mode using Thermo XCalibur software (v4.1.50). Full scan mass spectra (m/z 350 to m/z 1800) were acquired in the Orbitrap using an AGC target of 4 $x10^5$, a maximum injection time of 50 ms with a resolution of 120 000 (FWHM). Lock mass on the m/z 445.12003 siloxane ion was used for internal calibration. Each MS scan was followed by acquisition of fragmentation MS^2 spectra of the most intense ions for a total cycle time of 3 seconds (top speed mode). The selected ions were isolated using the quadrupole analyzer width of 1.6 Da and fragmented by Higher energy Collision-induced Dissociation (HCD) with 35% of collision energy. The resulting fragments were detected by the linear ion trap in rapid scan rate with an AGC target of $1x10^4$ and a maximum injection time of 50 ms. Dynamic exclusion of previously fragmented peptides was set for a period of 30 sec and a tolerance of 10 ppm.

Protein identification and data analysis

Mass spectra were searched against the Uniprot *Equus caballus* database (UniProt Reference Proteome – Proteome ID UP000002281– 44484 entries – 2021.05) using the search engine Andromeda integrated into the MaxQuant software (version 2.0.2.0) assuming the digestion enzyme trypsin. Cysteine carbamidomethylation was set as fixed modification and protein N-terminal acetylation and methionine oxidation were set as variable modifications. For protein validation, a false discovery rate (FDR) of 1% was allowed at peptide and protein level based on a target/decoy search. Label-free quantification (LFQ) was done with MaxQuant using a minimum of two unique peptides.

Text files generated by MaxQuant were analyzed using the R software (version 3.6.1). Only regular intensities from the proteinGroups.txt file were considered for data processing. Decoy proteins and potential contaminants were excluded from the analysis. The determination of a normalization factor was calculated for each sample by dividing their median intensity by the median of all the median intensities. For each sample, a noise value corresponding to the 0.01 percentile of all intensities of said sample was calculated and was imputed when an intensity value was missing from a sample. Only proteins identified with at least two razor unique peptides were considered as quantified proteins and kept in the analysis. The average intensity values from each experimental condition were calculated for each protein. Proteins with a ≥ 2-fold difference between the different conditions were analyzed with repeated two-Way ANOVA (with the disease status and the cell treatment with or without LPS as the independent variables) and were

considered significantly regulated with a $p < 0.05$. Data were not analyzed when a protein was not quantified in a sample from more than one condition. The human orthologs of differentially regulated proteins were searched with the Uniprot Database; an ortholog protein could not be found for 14 equine proteins that were not used in the gene ontology analysis.

Gene ontology was evaluated with Metascape using the GO Biological processes and Reactome Databases. Briefly, the Metascape online platform [5] identify gene ontology terms significantly enriched within a provided list of proteins. Statistical analysis includes corrections with the Benjamini-Hochberg tests for multiple comparisons. The software Cytoscape [6] (version 3.9.1) with the ClueGo [7] (version 2.5.9) and Cluepedia[8] (version 11.0_9606) plugins was used to visualize protein-protein interactions networks using preselected functions (gene ontology and Reactome pathways identified by Metascape), automatic parameters, yFiles Organic Layouts and the option connecting parent nodes up to the root node with Cluepedia.

Airway smooth muscle isolation and characterization

Cell culture

The endobronchial biopsies collected from each horse were kept in transport medium at 4 ^{o}C until processing within 60 minutes. Enzymatic digestion was performed as previously described [9]. Briefly, the biopsies were immersed for three hours in a digestion medium (Dulbecco's Modified Eagle Medium (DMEM)/F12 nutrient mix (Thermofisher, Waltham, MA) with 0.125 U/mL Collagenase H (Roche Diagnostics, Indianapolis, USA), 1 mg/mL Trypsin inhibitor (Sigma Aldrich, St. Louis, MO), 1 U/mL elastase (Worthington Biochemical, Lakewood, NJ), 1% Penicillin-Streptomycin (Wisent Inc., Saint-Jean-Baptiste, QC) and 0.1% Fungizone (Fisher Scientific, Hampton, NH). Then, the digested cells were seeded (50 000 cells/cm^3) into ventilated cell culture flasks with DMEM/F12 (3:1) medium (Life Technologies), supplemented with adenine 2.4 mg/L, 10% fetal bovine serum (FBS), 1% penicillin-streptomycin and 0.1% fungizone. The medium was changed every 24 h for the first 3 days and then every 48 h.

Flow cytometry

The purity of each ASM cell culture at the end of P4 was assessed by flow cytometry using specific contractile proteins (anti-α-SMA and anti-desmin) as previously described [9]. Briefly, ASM cells were counted, washed in PBS1X twice, and fixed for 20 min with 4% paraformaldehyde and then washed three

times in PBS 1X and kept at 4 ^{o}C for no longer than a month prior to analysis. After non-specific blocking with 5% normal horse serum and permeabilization with Cytofix-cytoperm, ASM cells (10^6) were stained for anti-α-SMA (mouse IgG2a, Sigma Aldrich, St. Louis, MO, 1:250) and anti-desmin (rabbit polyclonal IgG, Abcam, Cambridge, UK, 1:200) antibodies for one hour. Then, cells were incubated in the dark for 40 min with fluorescent dye-conjugated anti-IgG antibodies. Isotype-matched control antibodies (mouse IgG2a and rabbit serum) were used as negative controls. Flow cytometry acquisition of 10,000 events was performed using CellQuest Pro software on a FACSCalibur instrument (BD Bioscience). Signals greater than those of the isotype controls were considered positive, and the mean percentage of positive cells were evaluated.

Supplementary Figures

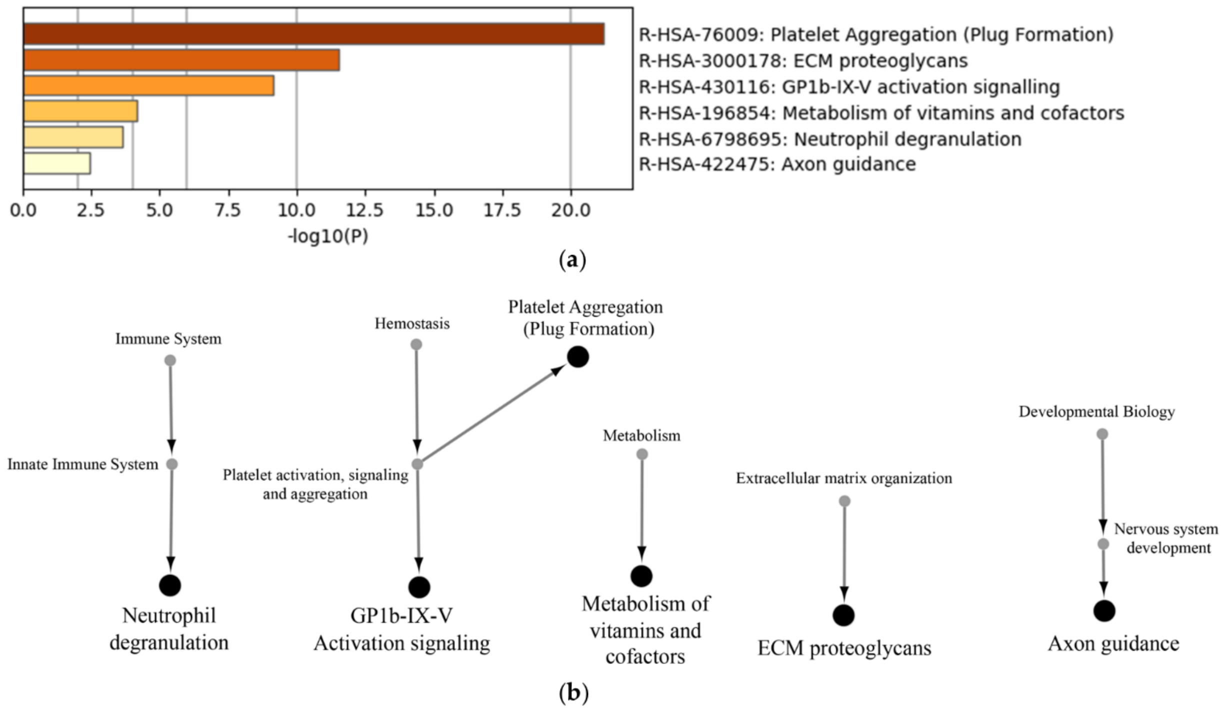

Supplementary Figure S1. Reactome enrichment analysis of proteins downregulated by LPS in neutrophil-derived EVs. Enrichment clusters identified by Metascape (**a**) and functional analysis of enriched GO terms from the parent node to the root node illustrated with ClueGo and Cluepedia in Cytoscape (**b**).

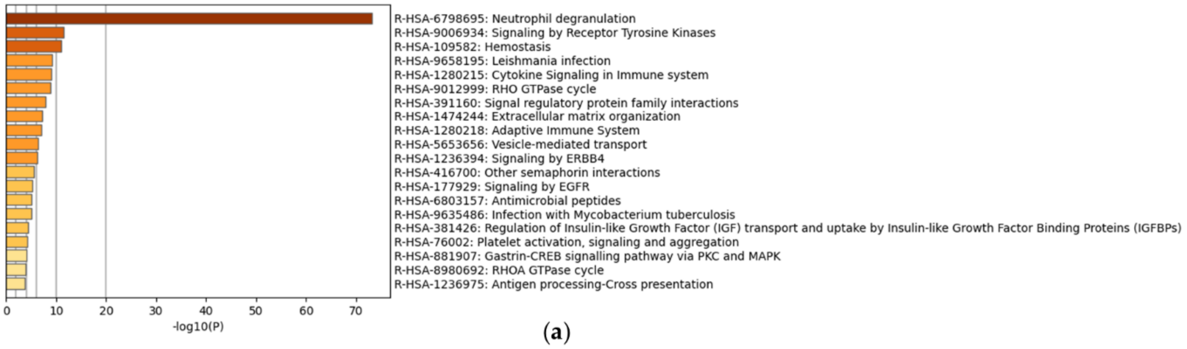

(**a**)

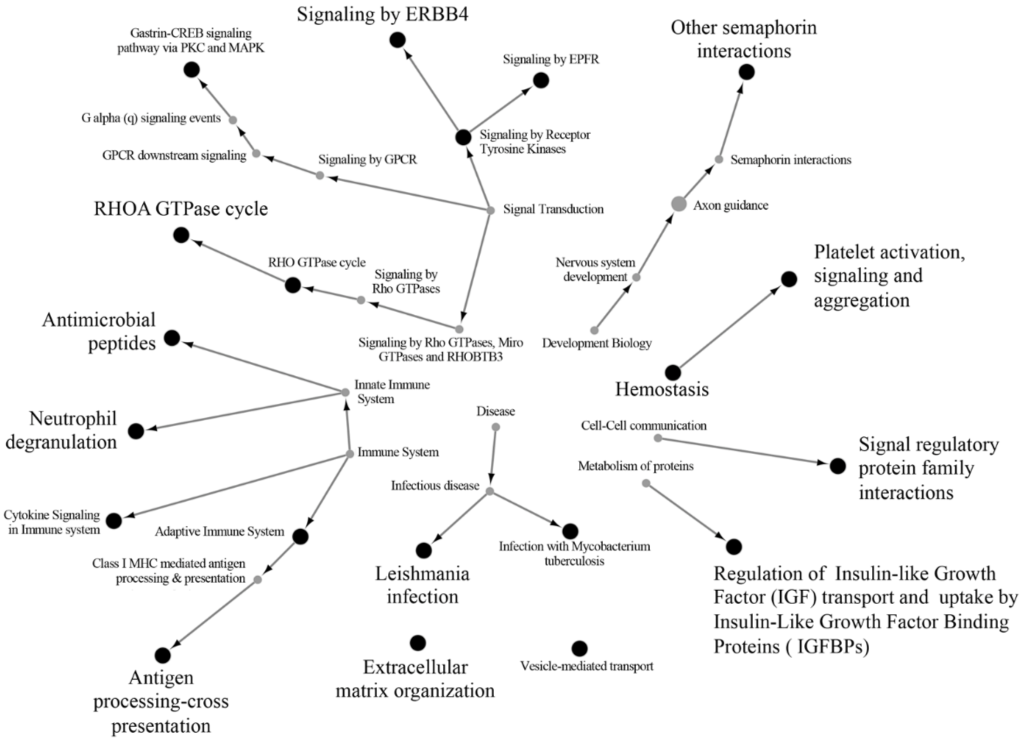

(**b**)

Supplementary Figure S2. Reactome enrichment analysis of proteins upregulated by LPS in neutrophil-derived EVs. Enrichment clusters identified by Metascape (**a**) and functional analysis of enriched GO terms from the parent node to the root node illustrated with ClueGo and Cluepedia in Cytoscape (**b**).

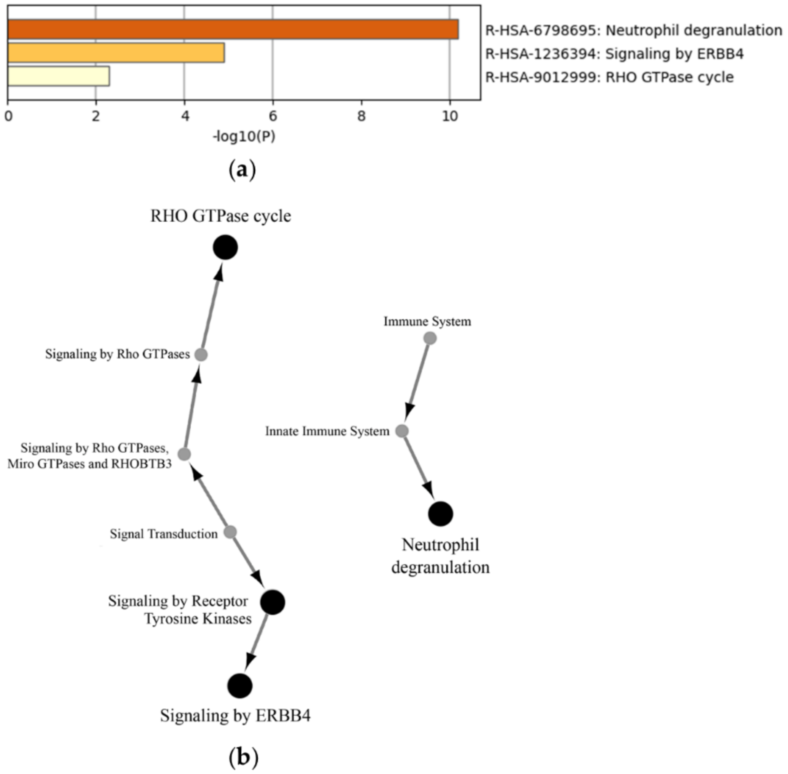

Supplementary figure S3. Reactome enrichment analysis of proteins upregulated by LPS in neutrophil-derived EVs only during asthmatic exacerbation. Enrichment clusters identified by Metascape (**a**) and functional analysis of enriched GO terms from the parent node to the root node illustrated with ClueGo and Cluepedia in Cytoscape (**b**).

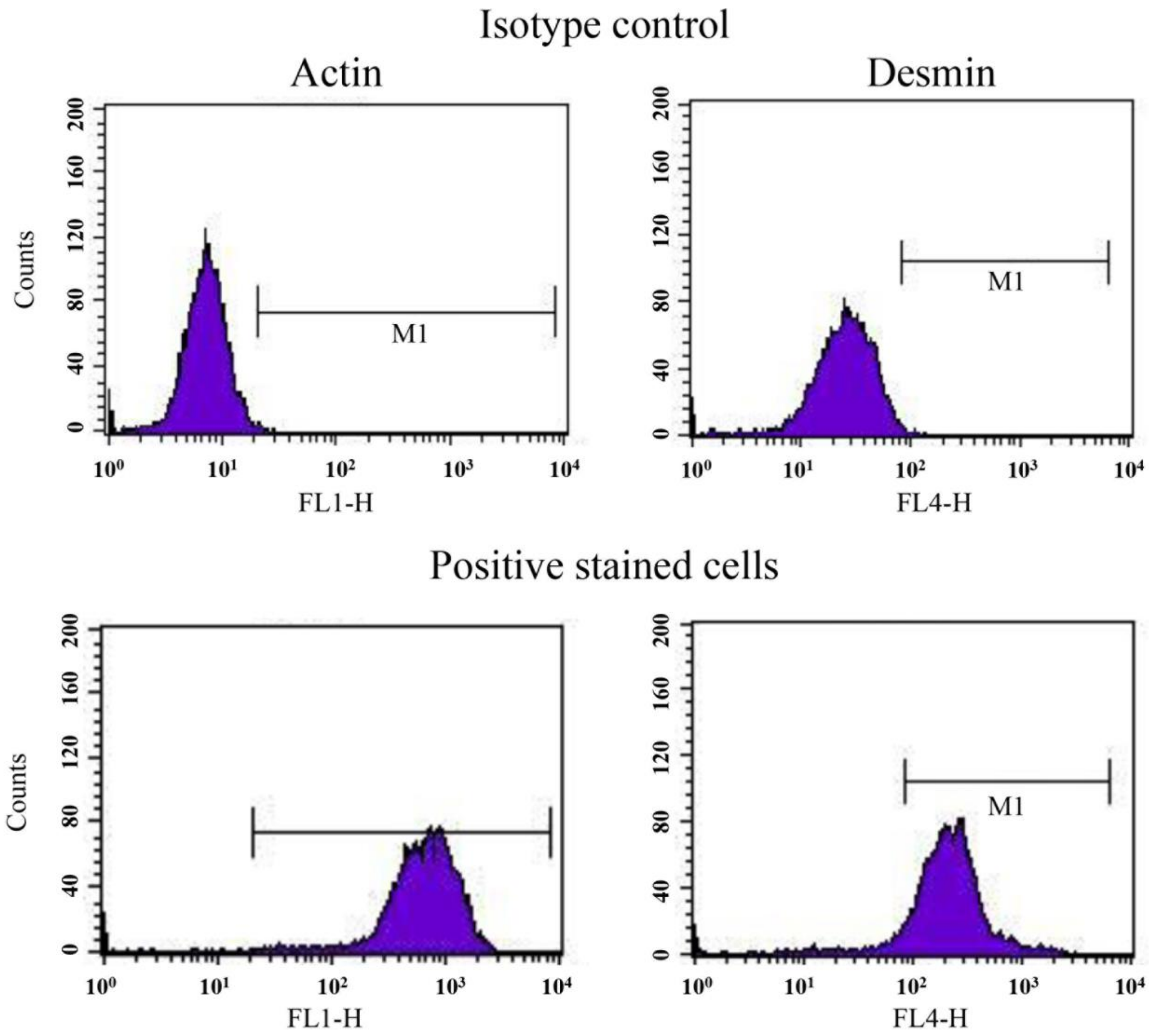

Supplementary Figure S4. Representative ASM cell flow cytometry analysis histograms for α-SMA (actin) and desmin. FL1-H = Intensity (height) in the FL1 channel (green fluorescence). FL4-H = Intensity (height) in the FL4 channel (red fluorescence). M1 = Histogram marker 1.

Supplementary tables

Supplementary table S1. Proteins significantly downregulated in EVs produced by LPS-stimulated neutrophils.

**Proteins downregulated by LPS in neutrophil-derived EVs**				
**Accession No.**	**Name**	**Human ortholog**	**p-value**	**Percentage of variation**
**F7ALC9**	Annexin A7	P20073	0.0074	45.3
**F7BAT4**	von Willebrand factor	P04275	0.0374	22.0
**A0A5F5Q281**	Collagen type I alpha 1 chain	P02452	0.0006	45.7
**A0A5F5PJM9**	Sorcin	P30626	0.0384	28.4
**F6WG72**	Penta-EF-hand domain containing 1	Q9UBV8	0.0074	40.3
**F7DE06**	Annexin	P50995	0.0063	51.1
**A0A5F5PV90**	Small nuclear ribonucleoprotein Sm D3	P62318	0.033	19.1
**A0A5F5PKJ8**	Signal transducing adaptor molecule	Q92783	0.0138	25.9
**A0A5F5PN66**	RNA-binding protein with serine-rich domain 1	Q15287	0.0198	46.0
**A0A3Q2HK18**	L-selectin	P14151	0.0304	26.5
**A0A3Q2KNS3**	Zinc finger DBF-type containing 2	Q9HCK1	0.0015	37.1
**F7CBT8**	Glucose transporter type 1, erythrocyte/brain	P11166	0.0234	7.5
**A0A3Q2H0K6**	ABC-type glutathione-S-conjugate transporter	P33527	0.0017	44.4
**F7B6G4**	Integrin subunit alpha 6	P23229	0.0123	13.2
**A0A5F5PR12**	Vanin 1	O95497	<0.0001	38.6
**A0A3Q2KM06**	Collagen type VI alpha 1 chain	P12109	0.0083	11.3
**F7APH4**	Osteonectin	P09486	0.0015	32.2
**F7CYR1**	Antithrombin-III	P01008	0.0009	30.7
**Q28369**	Retinol-binding protein 4	P02753	0.043	11.6
**F6Y5W8**	Rho guanine nucleotide exchange factor 5	Q12774	0.007	20.8
**A0A3Q2HL62**	Glycoprotein Ib platelet subunit beta	P13224	0.0167	17.2
**F7CN11**	Fibronectin	P02751	0.0262	19.8
**A0A3Q2H143**	Thromboxane A synthase 1	P24557	0.0084	21.1
**F6YUE1**	SPARC like 1	Q14515	0.0459	36.9
**A0A3Q2GXP0**	Grancalcin	P28676	0.0266	36.5
**A0A3Q2HNV2**	Insulin-like growth factor-binding protein 2	P18065	0.0134	11.4
**F6RTI8**	Collagen type I alpha 2 chain	P08123	0.0057	36.8
**F6TQF7**	Integrin subunit alpha 2b	P08514	0.0062	18.9
**F6RBT1**	Eosinophil peroxidase	P11678	0.0247	20.4
**A0A5F5PJQ9**	Fibrinogen beta chain	P02675	0.031	25.3
**A0A3Q2HJP2**	Integrin beta	P05106	0.0085	23.4
**P02062**	Hemoglobin subunit beta	P68871	0.0493	10.5
**F6W2Y1**	Fibrinogen gamma chain	P02679	0.0193	27.5

**A0A3Q2HTG2**	Fibrinogen alpha chain	P02671	0.0247	20.0
**F6XVR0**	Sulfotransferase	P50225	0.0375	33.9
**H9GZN9**	Uncharacterized protein	P01871	0.0321	12.4

Supplementary table S2. Proteins significantly upregulated in EVs produced by LPS-stimulated neutrophils.

**Proteins upregulated by LPS in neutrophil-derived EVs**				
**Accession No.**	**Name**	**Human ortholog**	**p-value**	**Percentage of variation**
**A0A3Q2KJ87**	Bactericidal permeability-increasing protein	P17213	0.0009	68.4
**F7B3H1**	Procollagen-lysine 5-dioxygenase	O60568	0.0032	60.3
**F6VS59**	Parvin gamma	Q9HBI0	0.0225	29.8
**F7DPD7**	Torsin	Q8N2E6	<0.0001	71.9
**A0A3Q2L464**	Multiple inositol-polyphosphate phosphatase 1	Q9UNW1	0.0025	43.5
**F6V0F7**	Hexosyltransferase	Q8N6G5	0.001	53.4
**F6VBP9**	Apolipoprotein E	P02649	0.0322	29.4
**A0A3Q2I4A5**	Elastase, neutrophil expressed	P08246	0.0018	65.4
**F7AED2**	Alpha-1-acid glycoprotein 2	P19652	0.0431	22.2
**F7CQ86**	Alpha-1-acid glycoprotein 2	P19652	0.0072	39.3
**A0A3Q2HNI5**	Glucosidase II alpha subunit	Q14697	0.0005	76.0
**A0A3Q2HWQ6**	Complement C3	P01024	0.0007	64.4
**F7CE24**	Tetraspanin	Q8NG11	0.0002	72.7
**F6TIR2**	Lipocalin 2	P80188	0.0026	51.4
**A0A5F5PES5**	Rho guanine nucleotide exchange factor 1	Q92888	0.0018	40.0
**F7BAR6**	Transmembrane 9 superfamily member	Q9HD45	0.0102	31.6
**F6W1S0**	Resistin	Q9HD89	0.0003	46.7
**F6PYT5**	Transmembrane channel-like protein	Q8IU68	0.0007	45.3
**A0A3Q2GT20**	Versican	P13611	0.0069	46.6
**A0A3Q2LEE0**	Protease, serine 57	Q6UWY2	0.0013	46.8
**F7DPZ6**	Olfactomedin 4	Q6UX06	0.0005	39.4
**F7C5U7**	DENN domain containing 3	A2RUS2	0.0087	36.8
**A0A3Q2I8H8**	Galectin-3-binding protein	Q08380	0.0368	42.5
**F6VFN4**	Sushi domain containing 3	Q96L08	0.0002	62.3
**A0A3Q2LDY0**	Syntaxin 2	P32856	0.0038	41.6
**F7E191**	Phospholipid-transporting ATPase	Q8TF62	0.0079	29.7
**F6XKR9**	Late endosomal/lysosomal adaptor and MAPK and MTOR activator 1	Q6IAA8	0.0088	43.0
**F6Y9M7**	C-X-C chemokine receptor type 4	P61073	0.0011	41.9
**F6TZ61**	Aldehyde dehydrogenase	P43353	0.0083	46.8
**F7CUM5**	Calcium and integrin binding 1	Q99828	0.002	38.9
**A0A3Q2HS16**	ADAM metallopeptidase domain 8	P78325	0.0026	35.3
**A0A3Q2HJN8**	Interleukin-1	P18510	0.0025	66.6
**F6T7K7**	Transcobalamin 1	P20061	0.0016	50.5
**F7E4J9**	Synaptotagmin like 1	Q8IYJ3	0.004	44.4
**F7D6J0**	Myeloid associated differentiation marker	Q96S97	0.006	35.6

**F6Y4R0**	Thioredoxin related transmembrane protein 1	Q9H3N1	0.0081	29.6
**A0A3Q2HBT4**	HORSE Polypeptide N-acetylgalactosaminyltransferase	Q10471	0.0093	55.3
**F7C456**	CD177 antigen	Q8N6Q3	0.0004	55.9
**A0A3Q2H4P6**	Carcinoembryonic antigen-related cell adhesion molecule 1	P13688	0.0006	32.5
**A0A3Q2I5A5**	Beta-1,4-galactosyltransferase 1	P15291	0.0048	52.2
**F7ADT4**	N-formyl peptide receptor 2	P25090	0.0014	42.1
**F6YZ58**	Solute carrier organic anion transporter family member	Q6ZQN7	0.0216	33.2
**F6WUK1**	ArfGAP with RhoGAP domain, ankyrin repeat and PH domain 1	Q96P48	0.0369	24.5
**F6USJ2**	Phospholipid-transporting ATPase	Q9Y2Q0	0.0124	37.4
**Q9MYW3**	Toll-like receptor 4	O00206	0.0004	42.1
**F7DTB6**	Karyopherin subunit beta 1	Q14974	0.0008	59.6
**F7AGL5**	92 kDa gelatinase (MMP9)	P14780	0.0067	53.7
**F6UG91**	Golgin A7	Q7Z5G4	0.0027	48.4
**A0A5F5PKL6**	Cathepsin G	P08311	0.0009	75.5
**H9GZT7**	Extended synaptotagmin 2	A0FGR8	0.0459	15.5
**F6SCH8**	Target of myb1 membrane trafficking protein	O60784	0.0008	45.2
**A0A3Q2GTF2**	Carcinoembryonic antigen-related cell adhesion molecule 1	P13688	<0.0001	51.2
**F6T1X0**	Carcinoembryonic antigen-related cell adhesion molecule 1	P13688	0.0009	45.2
**F6XN76**	E3 ubiquitin-protein ligase	Q96J02	0.002	49.5
**F6R7B4**	Matrix metallopeptidase 1 (MMP1)	P03956	0.0461	31.7
**A0A3Q2GS71**	Alpha-1-acid glycoprotein 2	P19652	0.0266	24.9
**A0A5F5Q329**	Ribosomal protein S6 kinase	Q15349	0.0051	21.5
**F6QGF7**	DnaJ heat shock protein family (Hsp40) member	Q9H3Z4	0.0001	44.5
**F7BKI9**	Toll interacting protein	Q9H0E2	0.0032	55.2
**O77811**	Lactotransferrin (Fragment)	P02788	0.0003	56.1
**A0A3Q2GSF8**	Nucleosome assembly protein 1 like 1	P55209	0.0335	5.7
**A0A5F5PT74**	Paxillin	P49023	0.0126	30.8
**A0A5F5PJ10**	Drebrin like	Q9UJU6	0.0130	32.1
**A0A3Q2HVS2**	RAB11 family interacting protein 1	Q6WKZ4	0.028	17.0
**F7B4U0**	Disco interacting protein 2 homolog B	Q9P265	0.0005	39.9
**F6U6V3**	Disintegrin and metalloproteinase domain-containing protein 10	O14672	0.0004	59.6
**F6XWM5**	Haptoglobin	P00738	0.0017	34.2
**A0A3Q2H2V4**	HECT-type E3 ubiquitin transferase	P46934	0.0063	21.4
**F7BTZ8**	Atlastin GTPase 3	Q6DD88	0.0302	25.8
**F6YTR6**	Rhomboid 5 homolog 2	Q6PJF5	0.0038	41.4
**F6U8J5**	Sialic acid-binding Ig-like lectin 5	O15389	0.0045	52.6

**A0A3Q2GWA2**	Thioredoxin interacting protein	Q9H3M7	0.0102	35.3
**F7D1W9**	Ficolin-1	O00602	0.0148	47.5
**F6RIG2**	ADP ribosylation factor like GTPase 8B	Q9NVJ2	0.0262	26.0
**A0A3Q2HG96**	ADAM metallopeptidase domain 17	P78536	0.0029	40.1
**A0A3Q2HGY5**	Tyrosine-protein kinase	P41240	0.0137	26.1
**A0A3Q2GTT1**	Ribosomal protein S6 kinase	Q15418	0.0398	13.0
**F7B7Q0**	Dedicator of cytokinesis 2	Q92608	0.01	20.9
**A0A3Q2I8S7**	Interferon-induced protein with tetratricopeptide repeats 1B	Q5T764	0.0059	44.4
**F6YTU4**	Docking protein 3	Q7L591	0.0111	27.4
**F7CGD4**	Non-specific protein-tyrosine kinase	Q14289		
**A0A3Q2GSP8**	Signal regulatory protein alpha	P78324	0.0011	20.18
**F7DB64**	Cytochrome b-245 light chain	P13498	0.0375	16.7
**F6U8Z5**	Calcium binding protein 39	Q9Y376	0.0015	35.8
**A0A3Q2HZX9**	Azurocidin 1	P20160	0.0028	41.2
**A0A3Q2I177**	Pleckstrin homology domain containing O2	Q8TD55	0.0008	30.3
**A0A3Q2HVB6**	Complement component 4 binding protein alph	P04003	0.0106	49.6
**F6ZNQ1**	Regulator of G protein signaling 14	O43566	0.0013	19.9
**F6QXT2**	VAMP associated protein A	Q9P0L0	0.0118	13.7
**F7B7M3**	Inositol 1,4,5-trisphosphate receptor-interacting protein	Q8IWB1	0.0137	28.5
**A0A3Q2LPJ8**	Adhesion G protein-coupled receptor E1	Q14246	0.0004	49.8
**A0A3Q2I7B5**	Mitogen-activated protein kinase kinase kinase kinase 4	O95819	0.0035	40.9
**F6U8F1**	Formin like 1	O95466	<0.0001	45.8
**F6RBQ4**	Ectonucleoside triphosphate diphosphohydrolase 3	O75355	0.0002	26.4
**F6WSK2**	Sialic acid-binding Ig-like lectin 5	O15389	<0.0001	45.3
**F7AT17**	Tyrosine-protein kinase	P08631	0.0002	47.8
**A0A5F5PWD5**	Serine/threonine kinase 26	Q9P289	0.0191	12.7
**A0A3Q2I8D8**	Glia-derived nexin	P07093	0.0053	53.3
**A0A5F5PG05**	Integrin beta	P05107	<0.0001	44.2
**F6WQL1**	Ecotropic viral integration site 2B	P34910	0.0232	12.7
**F7A0H6**	Folate receptor beta	P14207	0.0002	46.1
**F7AA00**	GRB2 binding adaptor protein, transmembrane	Q8N292	0.0002	29.5
**F7DA90**	Cytochrome b-245 beta chain	P04839	<0.0001	47.9
**F7AZ73**	Syntaxin binding protein 2	Q15833	0.0077	26.5
**F6QC17**	V-set immunoregulatory receptor	Q9H7M9	0.0063	21.5
**A0A5F5PLR4**	2,3-cyclic-nucleotide 3-phosphodiesterase	P09543	0.004	30.8
**F6TLH4**	Secretory carrier-associated membrane protein	O15127	0.0049	34.7

**A0A5F5PF14**	Brain abundant membrane attached signal protein 1	P80723	0.0001	21.3
**F6PUW3**	Maltase-glucoamylase	O43451	<0.0001	27.0
**A0A3Q2HN26**	Pleckstrin	P08567	0.0004	38.7
**F7CK20**	Tyrosine-protein kinase	P09769	0.0006	46.4
**F7BQS3**	Growth factor receptor bound protein 2	P62993	0.0042	33.8
**A0A3Q2IBB5**	Golgi apparatus protein 1	Q92896	0.0128	35.0
**F7BBA7**	Solute carrier family 44 member 2	Q8IWA5	0.0004	39.4
**F6RA08**	Stomatin	P27105	0.0297	22.4
**F6XAB0**	Synaptosomal-associated protein	O00161	0.0014	33.5
**F7ASL2**	DNAX-activation protein 12	O43914	0.0003	23.8
**F6QRY3**	Peptide-methionine (S)-S-oxide reductase	Q9UJ68	0.0047	37.6
**F6PM31**	Leukocyte specific transcript 1	O00453	0.0008	20.7
**A0A3Q2L513**	Sialic acid-binding Ig-like lectin 14	Q08ET2	0.0005	36.8
**F6WA92**	RAB5B, member RAS oncogene family	P61020	0.0019	30.4
**F7AQZ6**	Tyrosine-protein kinase	P07948	0.0004	47.0
**F6Z904**	RAB5A, member RAS oncogene family	P20339	0.0005	26.7
**A0A5F5PWB4**	Tyrosine-protein phosphatase non-receptor type	P29350	<0.0001	48.2
**F6RL28**	Protein XRP2	O75695	0.0003	42.9
**F6YW90**	Urokinase plasminogen activator surface receptor	Q03405	0.0043	29.0
**A0A5F5Q058**	Pleckstrin homology domain containing B2	Q96CS7	0.014	33.7
**F6Y9X0**	Hydrogen voltage-gated channel 1	Q96D96	0.0004	43.7
**A0A3Q2HX75**	Semaphorin 7A	O75326	0.0153	29.6
**F6PNU9**	ADP-ribosylation factor 6	P62330	<0.0001	38.4
**A0A5F5PRD3**	Non-specific serine/threonine protein kinase	O94804	0.0032	17.7
**A0A3Q2GZC0**	Cysteine rich secretory protein LCCL domain containing 2	Q9H0B8	0.0021	49.2
**F6QK77**	Deoxyribonuclease	P49184	<0.0001	36.1
**A0A3Q2LJE9**	Nicastrin	Q92542	0.0124	17.1
**A0A3Q2GYF6**	Protein-tyrosine-phosphatase	P08575	<0.0001	32.4
**F6S0P5**	ADP ribosylation factor 4	P18085	0.0368	29.5
**F7DGE2**	Vesicle-fusing ATPase	P46459	0.0208	22.3
**F6QUD4**	Wiskott-Aldrich syndrome protein family member	Q9Y6W5	0.0021	29.2
**A0A3Q2HC74**	Plexin C1	O60486	0.0002	28.4
**F7CEP4**	G protein-coupled receptor kinase	P43250	0.0027	23.6
**F6PM70**	Arachidonate 5-lipoxygenase	P09917	0.0061	28.1
**F6U4Y1**	TNF alpha induced protein 8	O95379	0.0035	16.6
**A0A3Q2IDH0**	CMRF35-like molecule 1	Q8TDQ1	0.0488	23.2
**F6SP02**	14-3-3 protein theta	P27348	0.0203	37.6
**F6X8Y3**	GNAS complex locus	P63092	0.0015	32.0

**F6VWQ7**	Thymocyte selection associated family member 2	Q5TEJ8	0.0121	27.1
**F6YR57**	Vesicle associated membrane protein 8	Q9BV40	0.0163	33.9
**F6R869**	Pre-mRNA processing factor 38B	Q5VTL8	0.0014	22.5
**A0A3Q2I008**	Ras-related protein Rap-2	P61225	0.0008	49.6
**A0A5F5PWD2**	RAB18, member RAS oncogene family	Q9NP72	0.0179	25.2
**F6YSK7**	Myristoylated alanine rich protein kinase C substrate	P29966	0.0116	20.5
**A0A3Q2HV04**	Reticulon	Q9NQC3	0.0146	30.7
**F6VYP4**	Abl interactor 1	Q8IZP0	0.0012	19.3
**F7B9Q9**	Protein kinase C and casein kinase substrate in neurons 2	Q9UNF0	0.0037	19.8
**A0A3Q2GTT9**	Macrophage-capping protein	P40121	0.0003	18.9
**F6TGW2**	Histidine ammonia-lyase	P42357	0.0489	22.7
**A0A3Q2HM94**	Solute carrier family 2 member 3	P11169	0.0001	37.1
**F6PSF7**	Triggering receptor expressed on myeloid cells 1	Q9NP99	0.0003	39.0
**F6QB61**	Peptidoglycan-recognition protein	O75594	0.0041	32.9
**F7B1Q3**	N-formyl peptide receptor 2-like	P25090	0.0004	44.2

Supplementary table S3. Proteins significantly upregulated in EVs produced by LPS-stimulated neutrophils, only during asthmatic exacerbation.

**Proteins upregulated by LPS only or more during the exacerbation phase of the disease in neutrophil-derived EVs**				
**Accession No.**	**Name**	**Human ortholog**	**p-value***	**Percentage of variation***
**F6WZ50**	Splicing factor 3b subunit 1	O75533	0.0034	4.3
**F7E0S8**	Regulator of G protein signaling 19	P49795	0.0151	13.0
**F6T0R5**	V-type proton ATPase subunit a	Q13488	0.0179	3.8
**A0A3Q2I8C1**	Phosphoglucomutase 2	Q96G03	0.0075	16.5
**A0A3Q2HG96**	ADAM metallopeptidase domain 17	P78536	0.0135	5.5
**F6WQL1**	Ecotropic viral integration site 2B	P34910	0.0067	9.2
**F6YTU4**	Docking protein 3	Q7L591	0.042	14.1
**A0A3Q2I427**	Leucine rich alpha-2-glycoprotein 1	P02750	0.0286	15.7
**F7CUM5**	Calcium and integrin binding 1	Q99828	0.0259	1.1
**F6U8J5**	Sialic acid-binding Ig-like lectin 5	O15389	0.0176	6.8
**F7B7Q0**	Dedicator of cytokinesis 2	Q92608	0.0422	3.4
**A0A3Q2I7B5**	Mitogen-activated protein kinase kinase kinase kinase 4	O95819	0.003	9.7
**F6U8F1**	Formin like 1	O95466	0.0269	4.4
**F7BQS3**	Growth factor receptor bound protein 2	P62993	0.0455	5.1
**F6QRY3**	Peptide-methionine (S)-S-oxide reductase	Q9UJ68	0.0491	4.8
**F6WA92**	RAB5B, member RAS oncogene family	P61020	0.0364	3.3
**F7AQZ6**	Tyrosine-protein kinase	P07948	0.0334	1.7
**F6RL28**	Protein XRP2	O75695	0.0317	1.9
**A0A3Q2LJE9**	Nicastrin	Q92542	0.0115	2.9
**A0A3Q2HC74**	Plexin C1	O60486	0.0469	2.7
**F7CEP4**	G protein-coupled receptor kinase	P43250	0.0143	0.81
**A0A3Q2I008**	Ras-related protein Rap-2	P61225	0.0244	3.5
**A0A3Q2GTT9**	Macrophage-capping protein	P40121	0.0038	20.9
**F6YSK7**	Myristoylated alanine rich protein kinase C substrate	P29966	0.0189	5.5
*** p-value and % of variation of the factor interaction of the disease status and cell treatment**				

Supplementary table S4. Biological processes enriched in proteins from neutrophil-derived EVs stimulated with LPS.

**Biological processes enriched in proteins from neutrophil-derived EVs stimulated with LPS**		
**Downregulation by LPS**		
**Biological process**	**Description**	**Proteins involved (symbol)**
**GO:0062023**	Collagen-containing extracellular matrix	ANXA7, ANXA11, SERPINC1, COL1A1, COL1A2, COL6A1, FGA, FGB, FGG, FN1, SPARC, VWF, SPARCL1
**GO:0031091**	Platelet alpha granule	FGA, FGB, FGG, FN1, ITGA2B, ITGB3, SPARC, VWF, GP1BB, HBB, SERPINC1, COL1A1, SLC2A1, ITGA6, SELL, SPARCL1, VNN1, EPX, GCA, ANXA7, TBXAS1, RBP4, SRI, COL1A2, ABCC1, ANXA11, IGFBP2, PEF1, STAM, ARHGEF5, SULT1A1
**GO:0002020**	Protease binding	SERPINC1, COL1A1, COL1A2, FN1, ITGB3, SELL, SRI, VWF, SULT1A1
**GO:0019838**	Growth factor binding	COL1A1, COL1A2, COL6A1, IGFBP2, ITGA6, ITGB3, ANXA7, FN1, ITGA2B, VWF, SPARC, SPARCL1, GP1BB, RBP4
**GO:0030667**	Secretory granule membrane	ANXA7, ITGA2B, ITGB3, SELL, SPARC, SRI, VNN1
**GO:0043589**	Skin morphogenesis	COL1A1, COL1A2, ITGA6, COL6A1, GP1BB, RBP4, ABCC1
**GO:0005509**	Calcium ion binding	ANXA7, ANXA11, SELL, SPARC, SRI, SPARCL1, GCA, PEF1
**GO:0090482**	Vitamin transmembrane transporter activity	ABCC1, RBP4, SLC2A1, VNN1, FN1, IGFBP2
**GO:0006979**	Response to oxidative stress	COL1A1, HBB, ABCC1, EPX, VNN1, TBXAS1
**GO:0050900**	Leukocyte migration	ITGA6, SELL, ARHGEF5, EPX
**GO:0032526**	Response to retinoic acid	COL1A1, IGFBP2, RBP4, ABCC1, SULT1A1, SLC2A1
**GO:0042470**	Melanosome	ANXA11, ITGB3, SLC2A1
**GO:0008289**	Lipid binding	ANXA7, ANXA11, RBP4, SELL, ARHGEF5, STAM
**GO:0042383**	Sarcolemma	COL6A1, SLC2A1, SRI
**GO:0005766**	Primary lysosome	ANXA11, VNN1, GCA
**GO:0050865**	Regulation of cell activation	FGG, FN1, GP1BB, IGFBP2, VNN1
**GO:0030139**	Endocytic vesicle	ANXA11, HBB, SPARC
**Upregulation by LPS**		
**GO:0042581**	Specific granule	ADAM8, ADAM10, ALDH3B1, CEACAM1, BPI, CYBA, CYBB, DNASE1L1, DOCK2, ELANE, STOM, FPR2, HP, ITGB2, KPNB1, LCN2, LTF, ORM2, PLAUR, PTPN6, RAP2B, SLC2A3, STK10, STXBP2,

		TCN1, VAMP8, SNAP23, PGLYRP1, TOM1, ATP8A1, OLFM4, TOLLIP, LAMTOR1, RETN, CD177, SLC44A2, ATP8B4, DNAJC5, TSPAN14, HVCN1, SLCO4C1, AZU1, B4GALT1, PTPRC, RAB5B, TYROBP, SIGLEC5, MGAM, VAPA, RAB18, NCSTN, DOK3, SIRPA, SIGLEC14, MMP9, DBNL, GOLGA7
**GO:0005766**	Primary lysosome	AZU1, BPI, C3, CTSG, ELANE, STOM, B4GALT1, ORM2, STXBP2, VAMP8, SNAP23, VAPA, TOM1, ATP8A1, NCSTN, TOLLIP, LAMTOR1, RETN, DNAJC5, SLCO4C1, PRSS57, ADAM8, VCAN, HCK, LYN, NSF, CXCR4, ARL8B, SLC44A2
**GO:0034774**	Secretory granule lumen	ALOX5, AZU1, BPI, C3, CTSG, DNASE1L1, DOCK2, ELANE, FCN1, FGR, HP, KPNB1, LCN2, LGALS3BP, LTF, ORM2, PTPN6, TCN1, PGLYRP1, OLFM4, DBNL, CAB39, TOLLIP, RETN, CRISPLD2, PRSS57, VCAN
**GO:0101002**	Ficolin-1-rich granule	ADAM8, ALOX5, FCN1, FPR2, ITGB2, KPNB1, MMP9, SLC2A3, SIGLEC5, MGAM, DBNL, CAB39, LAMTOR1, DOK3, PLEKHO2, CRISPLD2, SIRPA, SIGLEC14
**GO:0006954**	Inflammatory response	ADAM8, ALOX5, AZU1, C3, CYBA, CYBB, ELANE, FOLR2, FPR2, B4GALT1, HCK, HP, IL1RN, ITGB2, LYN, ORM2, TLR4, TYROBP, CXCR4, SEMA7A, SNAP23, THEMIS2, TREM1, TOLLIP, ITCH
**GO:0060627**	Regulation of vesicle-mediated transport	APOE, ARF6, AZU1, CEACAM1, C3, CSK, CYBA, DOCK2, FCN1, FGR, FPR2, HCK, ITGB2, LYN, NSF, PTPRC, RAB5A, RAB5B, STXBP2, VAMP8, PACSIN2, CD177, DNAJC5, SIRPA, CD300LF, ADAM8, ALOX5, ELANE, MMP9, PTPN6, TLR4, TYROBP, SEMA7A, PGLYRP1, RHBDF2, ITCH, IFIT1B, LTF, SERPINE2
**GO:0050865**	Regulation of cell activation	ADAM8, APOE, CEACAM1, BPI, CSK, CTSG, FGR, ITGB2, LYN, SERPINE2, PLEK, PTPN6, PTPRC, RPS6KA1, STXBP2, TLR4, TYROBP, LST1, VAMP8, PGLYRP1, THEMIS2, CD177, VSIR, ITCH, SIRPA, CD300LF, ADAM10, ALOX5, AZU1, ELANE, PTK2B, IL1RN, PLAUR, CXCR4, MAP4K4, PLXNC1, CIB1, OLFM4, MYADM, C4BPA, HCK, LTF, RHBDF2, C3, FCN1, SEMA7A, EVI2B, B4GALT1, RPS6KA2, ABI1
**GO:0002252**	Immune effector process	AZU1, C3, C4BPA, CTSG, DOCK2, ELANE, PTK2B, FCN1, FGR, HCK, LYN, PTPN6, STXBP2, ADAM17, TLR4, TYROBP, SNAP23, TREM1, ARL8B, GAPT, CSK, ADGRE1, DBNL
**GO:0031252**	Cell leading edge	ARF4, ARF6, CAPG, STX2, PTK2B, FGR, PLEK, PXN, RAB5A, ADAM17, TLR4, CXCR4, ABI1,

		WASF2, CIB1, PACSIN2, DBNL, CD177, MYADM, CEACAM1, B4GALT1, SYTL1
**GO:0030139**	Endocytic vesicle	ADAM8, APOE, ARF6, FMNL1, CYBA, CYBB, ELANE, HP, LTF, LYN, RAB5A, RAB5B, VAMP8, SNAP23, PGLYRP1, RAB11FIP1, HVCN1
**GO:1904724**	Tertiary granule lumen	HP, LTF, MMP9, PTPN6, TCN1, PGLYRP1, OLFM4, DBNL, GOLGA7, APOE
**GO:2000377**	Regulation of reactive oxygen species metabolic process	ALOX5, ARF4, CYBA, PTK2B, FPR2, GRB2, HP, ITGB2, TLR4, TYROBP, CD177, HVCN1, AZU1, FOLR2, LGALS3BP, APOE, KPNB1, CD300LF
**GO:0050900**	Leukocyte migration	ADAM8, ALOX5, AZU1, CTSG, ELANE, B4GALT1, HCK, ITGB2, LYN, MMP9, CXCR4, TREM1, CD177, SIRPA, DOCK2, FPR2, PLAUR, SEMA7A, PLXNC1
**GO:1903555**	Regulation of tumor necrosis factor superfamily cytokine production	ADAM8, AZU1, BPI, CYBA, CYBB, LTF, ORM2, PTPN6, PTPRC, TLR4, TYROBP, VSIR, SIRPA, C3, ELANE, FCN1, FGR, ADAM17, SEMA7A, CEACAM1
**GO:0001775**	Cell activation	ADAM10, AZU1, CTSG, DOCK2, PTK2B, FPR2, GNAS, ITGB2, LYN, PLEK, PTPN6, PTPRC, RAP2B, STXBP2, ADAM17, TLR4, TYROBP, SNAP23, NCSTN, TOLLIP, GAPT, HCK
**GO:0040017**	Positive regulation of locomotion	ADAM8, ADAM10, ARF6, AZU1, PTK2B, FGR, FPR2, LYN, MMP9, PTPRC, ADAM17, TLR4, CXCR4, SEMA7A, MAP4K4, ATP8A1, CIB1, RTN4, VSIR, MYADM, APOE, CEACAM1, CSK, ELANE, LTF, PTPN6, RAP2B, ABI1, RGS14, CAB39, GRB2, LAMTOR1, ITCH, SIRPA, C3, PLAUR
**GO:0001906**	Cell killing	AZU1, C3, CTSG, ELANE, LTF, PTPN6, STXBP2, PGLYRP1, TREM1, ARL8B, LYN, ADAM17, SNAP23, APOE, STOM, CXCR4, VAMP8, VAPA, ITCH, BPI, C4BPA, CNP, FGR, FPR2, HCK, HP, LCN2, TLR4, SIRPA, VCAN, SERPINE2, PTPRC, CRISPLD2, PRSS57, ALOX5, FCN1
**GO:0001558**	Regulation of cell growth	ADAM10, APOE, CEACAM1, CYBA, PTK2B, SERPINE2, RPS6KA1, ADAM17, CXCR4, SEMA7A, CIB1, DBNL, LAMTOR1, RTN4, DIP2B, ITCH, TMC8, BASP1, ARF4, CYBB, NEDD4, NSF, RAB5A, RGS14, SLC2A3
**GO:0005925**	Focal adhesion	ADAM10, ARF6, CYBA, PTK2B, HCK, ITGB2, MARCKS, PLAUR, PTPRC, PXN, ADAM17, SNAP23, MAP4K4, YWHAQ, PACSIN2, NCSTN, PARVG
**GO:0050778**	Positive regulation of immune response	ADAM8, C3, C4BPA, CSK, CTSG, ELANE, FCN1, FGR, FPR2, HCK, ITGB2, LYN, PTPN6, PTPRC, TLR4, TYROBP, VAMP8, THEMIS2, CD177, APOE, CEACAM1, CYBA, LTF, ITCH, IFIT1B, ADAM10,

		AZU1, PTK2B, ADAM17, CXCR4, CD300LF, RAB5A, ARL8B, FOLR2, STK26
**Only or more upregulated by LPS during exacerbation**		
**GO:0030667**	Secretory granule membrane	RAB5B, RAP2B, SIGLEC5, TCIRG1, NCSTN, DOK3, CAPG, RGS19, RP2
**GO:0070820**	Tertiary granule	RAP2B, SIGLEC5, TCIRG1, DOK3, LRG1, PGM2
**GO:0007264**	Small GTPase mediated signal transduction	DOCK2, GRB2, RAP2B, RGS19, DOK3
**GO:0070851**	Growth factor receptor binding	GRB2, LYN, ADAM17, NCSTN, RAP2B, RGS19, CAPG, DOK3, LRG1
**GO:0030097**	Hemopoiesis	DOCK2, EVI2B, LYN, ADAM17, TCIRG1, CIB1, RAP2B, NCSTN, FMNL1, RAB5B
**GO:0030099**	Myeloid cell differentiation	EVI2B, LYN, TCIRG1, CIB1, ADAM17, MAP4K4, PLXNC1, RAP2B, GRB2
**GO:0030036**	Actin cytoskeleton organization	FMNL1, CAPG, DOCK2, GRB2, MARCKS
**GO:0042581**	Specific granule	DOCK2, RAP2B, LRG1, PGM2
**GO:0005925**	Focal adhesion	MARCKS, ADAM17, MAP4K4, NCSTN
**GO:0001726**	Ruffle	CAPG, ADAM17, CIB1, MARCKS, MSRA, LYN
**GO:0031267**	Small GTPase binding	FMNL1, DOCK2, CIB1, PLXNC1
**GO:0016773**	Phosphotransferase activity, alcohol group as acceptor	GRK6, LYN, MAP4K4, PGM2
**GO:0030674**	Protein-macromolecule adaptor activity	GRB2, CIB1, NCSTN, CAPG, MARCKS
**GO:0006897**	Endocytosis	DOCK2, GRB2, RAB5B, LYN

References in Supplement

1. Brazil, T.J.; Dagleish, M.P.; McGorum, B.C.; Dixon, P.M.; Haslett, C.; Chilvers, E.R. Kinetics of pulmonary neutrophil recruitment and clearance in a natural and spontaneously resolving model of airway inflammation. *Clin Exp Allergy* **2005**, *35*, 854-865, https://doi:10.1111/j.1365-2222.2005.02231.x.
2. Dunkel, B.; Rickards, K.J.; Werling, D.; Page, C.P.; Cunningham, F.M. Neutrophil and platelet activation in equine recurrent airway obstruction is associated with increased neutrophil CD13 expression, but not platelet CD41/61 and CD62P or neutrophil-platelet aggregate formation. *Vet Immunol Immunopathol* **2009**, *131*, 25-32, https://doi:10.1016/j.vetimm.2009.03.004.
3. Koblinger, K.; Hecker, K.; Nicol, J.; Wasko, A.; Fernandez, N.; Leguillette, R. Bronchial collapse during bronchoalveolar lavage in horses is an indicator of lung inflammation. *Equine Vet J* **2014**, *46*, 50-55, https://doi:10.1111/evj.12096.
4. Douanne, N.; Dong, G.; Amin, A.; Bernardo, L.; Blanchette, M.; Langlais, D.; Olivier, M.; Fernandez-Prada, C. Leishmania parasites exchange drug-resistance genes through extracellular vesicles. *Cell Rep* **2022**, *40*, 111121, https://doi:10.1016/j.celrep.2022.111121.
5. Zhou, Y.; Zhou, B.; Pache, L.; Chang, M.; Khodabakhshi, A.H.; Tanaseichuk, O.; Benner, C.; Chanda, S.K. Metascape provides a biologist-oriented resource for the analysis of systems-level datasets. *Nat Commun* **2019**, *10*, 1523, https://doi:10.1038/s41467-019-09234-6.
6. Shannon, P.; Markiel, A.; Ozier, O.; Baliga, N.S.; Wang, J.T.; Ramage, D.; Amin, N.; Schwikowski, B.; Ideker, T. Cytoscape: a software environment for integrated models of biomolecular interaction networks. *Genome Res* **2003**, *13*, 2498-2504, https://doi:10.1101/gr.1239303.
7. Bindea, G.; Mlecnik, B.; Hackl, H.; Charoentong, P.; Tosolini, M.; Kirilovsky, A.; Fridman, W.H.; Pages, F.; Trajanoski, Z.; Galon, J. ClueGO: a Cytoscape plug-in to decipher functionally grouped gene ontology and pathway annotation networks. *Bioinformatics* **2009**, *25*, 1091-1093, https://doi:10.1093/bioinformatics/btp101.
8. Bindea, G.; Galon, J.; Mlecnik, B. CluePedia Cytoscape plugin: pathway insights using integrated experimental and in silico data. *Bioinformatics* **2013**, *29*, 661-663, https://doi:10.1093/bioinformatics/btt019.
9. Vargas, A.; Peltier, A.; Dube, J.; Lefebvre-Lavoie, J.; Moulin, V.; Goulet, F.; Lavoie, J.P. Evaluation of contractile phenotype in airway smooth muscle cells isolated from endobronchial biopsy and tissue specimens from horses. *Am. J. Vet. Res.* **2017**, *78*, 359-370, https://doi:10.2460/ajvr.78.3.359.
